# RNA biomarkers for alcohol use disorder

**DOI:** 10.3389/fnmol.2022.1032362

**Published:** 2022-11-04

**Authors:** Laura B. Ferguson, R. Dayne Mayfield, Robert O. Messing

**Affiliations:** ^1^Waggoner Center for Alcohol and Addiction Research, University of Texas at Austin, Austin, TX, United States; ^2^Department of Neurology, Dell Medical School, University of Texas at Austin, Austin, TX, United States; ^3^Department of Neuroscience, University of Texas at Austin, Austin, TX, United States

**Keywords:** biomarker, microarray, transcriptome, alcohol dependence, alcoholism, RNA seq

## Abstract

Alcohol use disorder (AUD) is highly prevalent and one of the leading causes of disability in the US and around the world. There are some molecular biomarkers of heavy alcohol use and liver damage which can suggest AUD, but these are lacking in sensitivity and specificity. AUD treatment involves psychosocial interventions and medications for managing alcohol withdrawal, assisting in abstinence and reduced drinking (naltrexone, acamprosate, disulfiram, and some off-label medications), and treating comorbid psychiatric conditions (e.g., depression and anxiety). It has been suggested that various patient groups within the heterogeneous AUD population would respond more favorably to specific treatment approaches. For example, there is some evidence that so-called reward-drinkers respond better to naltrexone than acamprosate. However, there are currently no objective molecular markers to separate patients into optimal treatment groups or any markers of treatment response. Objective molecular biomarkers could aid in AUD diagnosis and patient stratification, which could personalize treatment and improve outcomes through more targeted interventions. Biomarkers of treatment response could also improve AUD management and treatment development. Systems biology considers complex diseases and emergent behaviors as the outcome of interactions and crosstalk between biomolecular networks. A systems approach that uses transcriptomic (or other -omic data, e.g., methylome, proteome, metabolome) can capture genetic and environmental factors associated with AUD and potentially provide sensitive, specific, and objective biomarkers to guide patient stratification, prognosis of treatment response or relapse, and predict optimal treatments. This Review describes and highlights state-of-the-art research on employing transcriptomic data and artificial intelligence (AI) methods to serve as molecular biomarkers with the goal of improving the clinical management of AUD. Considerations about future directions are also discussed.

## Introduction

Alcohol use disorder (AUD) is highly prevalent and one of the leading causes of disability in the United States, second only to ischemic heart disease (World Health Organization [WHO], 2020). AUD is classified in the Fifth Edition of the Diagnostic and Statistical Manual of Mental Disorders as a syndrome of two or more of eleven behavioral symptoms with severity based on the number of symptoms present (American Psychiatric Association [APA], 2013): (1) Missing work or school, (2) Drinking in hazardous situations, (3) Drinking despite social or personal problems, (4) Craving for alcohol, (5) Buildup of tolerance, (6) Withdrawals when trying to quit, (7) Drinking more than intended, (8) Trying to quit without success, (9) Increased alcohol-seeking behavior, (10) Interference with important activities, (11) Continued use despite health problems. While there are some molecular biomarkers of heavy alcohol use and alcohol-related liver damage that can suggest AUD, e.g., γ-Glutamyl transferase (GGT), aspartate amino-transferase (AST), alanine amino-transferase (ALT), mean corpuscular volume (MCV), and carbohydrate-deficient transferrin (CDT) (Jastrzebska et al., 2016), these are lacking in diagnostic accuracy (Gough et al., 2015). Therefore, improved diagnostic biomarkers are needed. Currently, AUD treatment involves psychosocial interventions and medications for managing alcohol withdrawal, treating comorbid psychiatric conditions (e.g., depression and anxiety), and reducing drinking (naltrexone, acamprosate, disulfiram, and some off-label medications) (Winslow et al., 2016; Kranzler and Soyka, 2018; Ray et al., 2019; Witkiewitz et al., 2019; Mason and Heyser, 2021). It has been suggested that various patient groups within the heterogeneous AUD population could respond more favorably to specific treatment approaches (Mann et al., 2018). There have been attempts to behaviorally identify reward drinkers and relief drinkers for this purpose (Mann et al., 2018). However, there are currently no biomarkers to stratify patients into optimal treatment groups or to monitor and predict treatment response (refer to Table 1 for biomarker definitions). Additionally, the available pharmaceutical treatments for AUD are only moderately effective, and not effective for all patients (Winslow et al., 2016). To facilitate the drug development process, the ability to objectively monitor clinically relevant changes non-invasively are needed.

**TABLE 1:** Types of biomarkers.

1. Susceptibility/risk biomarker	Indicates the potential for developing a disease or medical condition in an individual who does not currently have clinically apparent disease or the medical condition.
2. Diagnostic biomarker	Detect or confirm presence of a disease or condition of interest or to identify individuals with a subtype of the disease.
3. Monitoring biomarker	Measured repeatedly for assessing status of a disease or medical condition or for evidence of exposure to (or effect of) a medical product or an environmental agent.
4. Prognostic biomarker	Identify likelihood of a clinical event, disease recurrence or progression in patients who have the disease or medical condition of interest.
5. Predictive biomarker	Identify individuals who are more likely than similar individuals without the biomarker to experience a favorable or unfavorable effect from exposure to a medical product or an environmental agent.
6. Response biomarker	Used to show that a biological response, potentially beneficial or harmful, has occurred in an individual who has been exposed to a medical product or an environmental agent (includes pharmacodynamic biomarkers and surrogate endpoint biomarkers).
7. Safety biomarker	Measured before or after an exposure to a medical product or an environmental agent to indicate the likelihood, presence, or extent of toxicity as an adverse effect.

Biomarker development and personalized medicine approaches have been identified as top priorities to expedite translational research in AUD (Litten et al., 2015; Heilig et al., 2016; Ray et al., 2021). To standardize the definitions and concepts governing the use of biomarkers in research and clinical practice, the FDA-NIH Biomarker Working Group (2016) created the Biomarkers, EndpointS, and other Tools (BEST) glossary which defines a biomarker as a defined characteristic that is measured as an indicator of normal biological processes, pathogenic processes, or responses to an exposure or intervention, including therapeutic interventions. There are seven categories of biomarkers (Table 1). Biomarkers can be derived from molecular, histologic, imaging (CT, PET, MRI, MEG), or physiologic characteristics (e.g., EEG). Molecular markers can include nucleic acids-based biomarkers such as gene mutations or polymorphisms, methylation status, and quantitative gene expression analysis (RNA), peptides, proteins, lipids, metabolites, and other small molecules.

**FIGURE 1:**
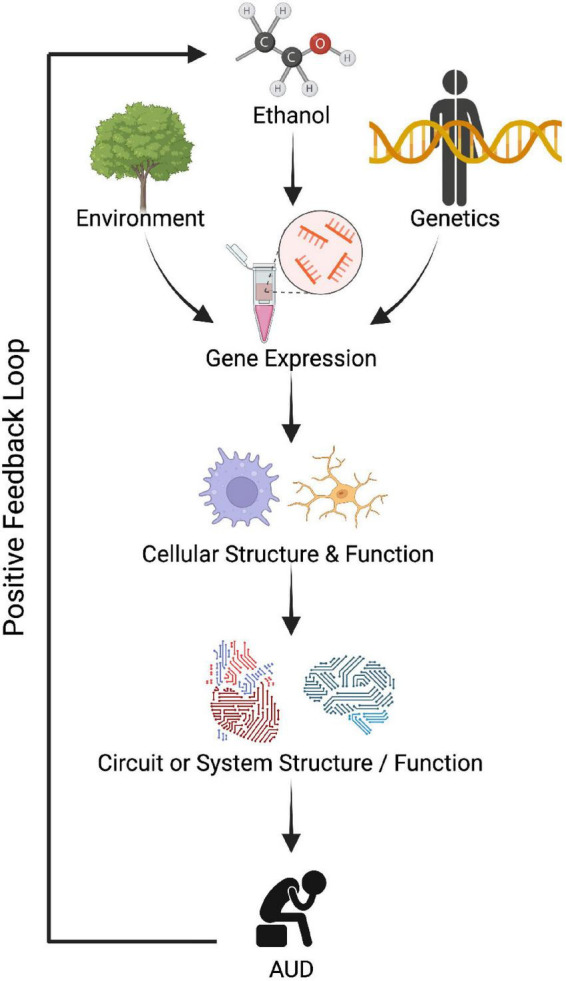
Dysregulated gene expression could lead to improper cellular function, circuit function, and eventually contribute to abnormal behavior observed in alcohol use disorder (AUD). Created with BioRender.com.

Alcohol use disorder is a complex disorder with both genetic and environmental factors that contribute in roughly equal proportions (e.g., Verhulst et al., 2015). Research efforts to identify the genetic variations associated with AUD, including many large genome-wide association studies (GWAS), have revealed that: (1) AUD is highly polygenic (with hundreds of variants across the genome), (2) the effect sizes of individual genetic variants are small, (3) many of the AUD-related variants are in non-coding areas of the genome, and (4) there is a large gap in understanding how genetic variation shapes AUD phenotypes (Clarke et al., 2017; Walters et al., 2018; Deak et al., 2019; Kranzler et al., 2019; Barr et al., 2020; Gupta et al., 2020; Sanchez-Roige et al., 2020; Zhou et al., 2020; Biernacka et al., 2021). For these reasons, it is increasingly recognized that intermediate phenotypes (e.g., transcriptomics, epigenomics, proteomics, and metabolomics) are important to bridge this gap as they can incorporate both genetic and environmental information (Figure 1; Mayfield and Harris, 2009; Liu et al., 2014; Lin et al., 2016; Zhang and Gelernter, 2017).

Technological advancements in next generation RNA sequencing and other omics technologies have positioned these intermediary molecules to be especially useful biomarkers for complex diseases like AUD. In addition to incorporating both genetic and environmental information, the detection methods are accurate, relatively easy to carry out and interpret, and inexpensive (compared to other modalities, e.g., functional magnetic resonance imaging). Furthermore, these technologies involve the measurement of many molecules simultaneously permitting systems-level molecular approaches and facilitating the selection of biomarker panels. While analyzing single targets is appealing for simplicity, expecting a single molecule to capture the state of a complex disorder like AUD is unrealistic. Instead, systems approaches are required for analysis of complex disease states resulting from network perturbations (Cho et al., 2012), and biomarker panels have been shown to outperform single biomarkers for diagnosing brain diseases, e.g., depression (Cuomo-Haymour et al., 2022b) and multiple sclerosis (Cuomo-Haymour et al., 2022a), and cancer (Muinao et al., 2019). There are several commercially available transcriptomic-based assays for cancer, such as Oncotype DX (Genomic Health), Mammaprint (Agendia), and Prosigna (Veracyte), that use panels of mRNAs to assess the prognosis and possible requirement of chemotherapy treatment in breast cancer patients (Duffy et al., 2016; Narrandes and Xu, 2018). One important lesson learned from these transcriptomics-based assays for cancer is that it usually requires a panel of biomarkers, a biomarker signature, to accurately predict prospective outcomes and achieve clinically actionable results (Cardoso et al., 2016; Schaafsma et al., 2021).

While systems approaches that employ transcriptomics are in clinical use for precision medicine in cancer (Narrandes and Xu, 2018), success has lagged for psychiatric and behavioral disorders given funding limitations and poor access to brain tissue, which is the primary dysfunctional organ in AUD and other mental illnesses (Kranzler et al., 2017). However, blood and saliva are easily obtained, routinely sampled in clinical practice through minimally invasive means, posing little harm to the patient. Importantly, blood- and saliva-derived biomarkers show promise for guiding the diagnosis, prognosis, or treatment for behavioral disorders and psychiatric illnesses (e.g., Munkholm et al., 2015; Hicks et al., 2018; Hess et al., 2020; Le-Niculescu et al., 2021; Wagh et al., 2021; see Supplementary Table 1 for more references). These examples demonstrate that molecular data in peripheral tissues can be clinically useful as biomarkers for psychiatric and behavioral disorders.

As -omics-based technologies have emerged, more studies have employed them to explore alcohol’s effects in peripheral tissues. The goal of this Review is to synthesize the literature on transcriptome-based biomarkers for AUD from accessible tissues. Challenges and future directions for RNA biomarkers are also discussed. The discussion of other omics-based intermediary phenotypes in peripheral tissues for use in AUD management is outside the scope of this Review, but interested readers are referred to articles regarding DNA methylation markers (Zhang H. et al., 2013; Zhang R. et al., 2013; Hagerty et al., 2016; Clark et al., 2018, 2022; Liu et al., 2018; Philibert et al., 2018; Wilson et al., 2019; Xu et al., 2019; Dugue et al., 2021; Liang et al., 2021; Miller et al., 2021), proteomic markers (Freeman et al., 2010, 2011; Waszkiewicz et al., 2014; Lai et al., 2015; Liangpunsakul et al., 2015; Batista et al., 2019; Niu et al., 2022) or metabolomic markers (Mittal and Dabur, 2015; Hinton et al., 2017; Mostafa et al., 2017; Karkkainen et al., 2020; Liu et al., 2022; Nahar et al., 2022).

## Methods

For this Review, the PubMed database was searched for relevant literature using a Boolean search strategy (inception through 2 July 2022): (microarray OR RNA seq OR transcriptome) AND (plasma OR blood OR serum OR saliva OR urine) AND (alcohol dependence OR alcohol abuse OR alcohol use disorder) NOT cancer NOT liver.

The articles were evaluated for the following inclusion characteristics: related to alcohol use disorder (studies on liver disease and carcinoma were excluded), genome-wide study (i.e., multiple biomarkers evaluated in a hypothesis-free fashion), some metric of biomarker performance (e.g., accuracy or area under the receiver operating characteristic curve), English language, full text availability. The references from the articles were also considered for inclusion in the Review. The included manuscripts were reviewed by the author to provide an overview of the current knowledge regarding transcriptome-based biomarkers in AUD.

## Results

Before detailing the results of the literature search, it is necessary to provide some definitions and background on the transcriptome and how biomarker performance is evaluated.

### Evaluating biomarker performance

Some background is provided here to give the reader a basic understanding of classifiers [a type of machine learning (ML)] and the evaluation of classifier performance. A classification problem can be approached using supervised and unsupervised methods. In contrast to unsupervised clustering, supervised classifiers learn a function from training data that consist of pairs of input objects (e.g., gene expression signatures of one or more genes) and desired outputs (e.g., AUD or non-AUD). The function learned will depend on the specific ML model the user selects. Some examples are Random Forest, Neural Network, Logistic Regression, Naïve Bayes, Support Vector Machines (SVMs), and others. Each has its own set of assumptions and tradeoffs in terms of model complexity, how interpretable the model is, and computational costs for training the model. An overview of the available classification techniques is beyond the scope of this Review but interested readers can refer to Libbrecht and Noble (2015), Kuhn (2018), and Starmer (2022).

Although a description of the available ML methods is outside the scope of this Review, we will briefly describe three popular classification methods that are used in two of the RNA biomarker studies discussed below (Rosato et al., 2019; Ferguson et al., 2022): Random Forest (RF), Logistic Regression (LR), and Partial Least Squares Discriminant Analysis (PLSDA). RF models combine the output of multiple decision trees to reach a single result. LR is like linear regression except that it fits a curve instead of a line to the data and predicts a discrete variable (class membership, e.g., AUD or non-AUD) instead of a continuous variable (e.g., lifetime ethanol consumption). As with linear regression, the coefficients are a measure of feature importance in LR. PLSDA is a “supervised” version of Principal Component Analysis (PCA) in that it reduces the dimensionality of the dataset with respect to class labels. The goal is to maximize the covariance between a linear combination of the genes and the class label. Each of these three techniques provide a measure of feature importance, so they can be used as classifiers with built-in feature selection. They can also be used as feature selectors to select the most important features to be fed into different classification algorithms [as done in a study discussed in Section “Artificial intelligence” below (Hahn et al., 2022)].

These classification techniques are a good starting place with transcriptomic datasets because they produce relatively interpretable models and include measures of feature importance which could offer biological insights into AUD based on the most differentiating features between the classes being distinguished. However, these are only three of many techniques, and it is difficult to determine what techniques will produce the highest performing models for a given dataset, *a priori*. It is advisable to try as many different methods as possible beginning with the simplest. This is because complex models do not necessarily outperform simpler models (for example, neural networks will not necessarily distinguish classes better than a RF), and there is a tradeoff between model complexity and interpretability/computational expense. The simpler the model the less computational load required to train the model and the more interpretable the final model will be.

There are several different metrics used to estimate the performance of a biomarker or panel of biomarkers in classifying samples as one class or another (e.g., AUD versus non-AUD, likely to benefit from a treatment versus not likely to benefit). Some of the most common are accuracy, sensitivity, specificity, and area under receiver operating characteristic curve (AUC). Once a classification model is constructed, its performance can be evaluated on an unseen data set (it is important that the data used to test the model’s performance was not involved in building the model). Given a test set and a classifier, each decision will be one of the following: (1) true positive (a positive example classified as positive), (2) false negative (a positive example misclassified as negative), (3) true negative (a negative example classified as negative), (4) false positive (a negative example misclassified as positive). A contingency table can be constructed from this information as a 2 × 2 matrix where the columns are the known true classes and the rows are the predicted classes. Several common metrics can be calculated from this table: Accuracy (the fraction of predictions the model assigned correctly): $\frac{T\,P+T\,N}{T\,P+T\,N+F\,P+F\,N}$, sensitivity or True Positive Rate (the proportion of positives correctly identified): $\frac{T\,P}{T\,P+F\,N}$, specificity or True Negative Rate (the proportion of negatives correctly identified): $\frac{T\,N}{T\,N+F\,P}$, and the False Positive Rate: $1-\frac{T\,N}{T\,N+F\,P}$. A ROC curve plots the True Positive Rate on the Y axis and the False Positive Rate on the X axis so that it provides a two-dimensional depiction of classifier performance as it depicts relative tradeoffs between the benefits (true positives) and costs (false positives) of a classifier under different classification thresholds (Linden, 2006). To summarize the two-dimensional graph into a single metric, the area under the curve (AUC) can be calculated. The higher the AUC, the better the classifier performed. AUC of 0.5 is random, 1.0 is perfect, and 0.7–0.8 is generally considered high performance (Crow et al., 2019).

### Transcriptome background

The transcriptome is the complete set of RNA transcripts produced by a genome which includes both protein-coding messenger RNAs (mRNAs) and non-coding RNAs (ncRNAs). The mRNA transcripts reflect which genes are actively expressed. mRNA levels can be compared between two conditions to identify differentially expressed genes (DEGs) pertaining to a biological state of interest (e.g., AUD versus non-AUD controls). The non-coding RNAs include both housekeeping RNAs (snRNA, tRNA, rRNA, snoRNA) and regulatory RNAs [microRNA (miRNA), small interfering RNA (siRNA), Piwi interacting RNAs (piRNA), long non-coding RNA (lncRNA), and circular RNA (circRNA)]. The transcriptome is affected by both genetic variation and environmental conditions (Figure 1). Microarray and next generation RNA sequencing (RNA seq) technologies have made it possible to measure the levels of all RNA molecules genome-wide and simultaneously, providing an exceptional opportunity for systems-level approaches.

Transcriptome studies in the alcohol research field have revealed that alcohol use affects the expression patterns of hundreds of mRNAs and ncRNAs in the brain tissue of humans and animal models, e.g., see reviews and primary research articles (Contet, 2012; Nunez and Mayfield, 2012; Mayfield, 2017; Warden and Mayfield, 2017; Ferguson et al., 2019; Brenner et al., 2020; Drake et al., 2020). In addition to brain tissue, some studies have explored alcohol’s effects in peripheral tissues including blood and saliva (Beech et al., 2012, 2014; Hicks et al., 2012; Kupfer et al., 2013; McClintick et al., 2014, 2019; Ignacio et al., 2015; Barr et al., 2016; Sureshchandra et al., 2016, 2019; Ten Berg et al., 2018; Rosato et al., 2019; Lewis et al., 2021; Liu et al., 2021; Mead et al., 2021; Ferguson et al., 2022; Gong et al., 2022). These studies will be detailed in the following sections.

### Literature search results overview

**FIGURE 2:**
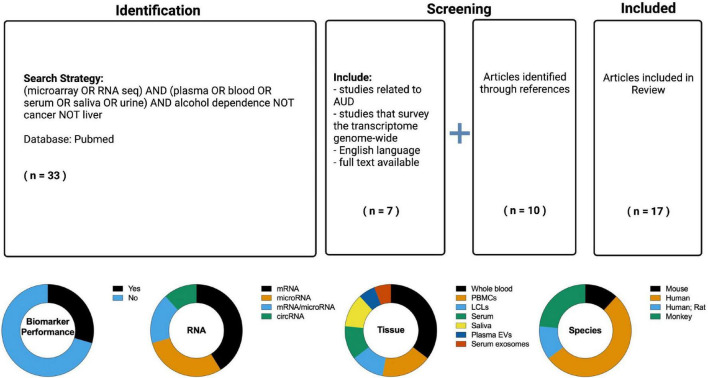
(Top) PubMed was searched (inception through 2 July 2022) for relevant literature using a Boolean search strategy. The articles were evaluated for the following inclusion characteristics: English language, full text availability, related to alcohol use disorder (the search results contained many studies related to liver diseases and carcinomas which were excluded), non-targeted study with multiple biomarkers evaluated, some metric of biomarker utility (e.g., accuracy, area under the receiver operating characteristic curve). References from articles in the search were also considered for inclusion in the Review. The resulting information from these searches was reviewed to provide an overview of the current knowledge regarding RNA based biomarkers in alcohol use disorder (AUD). (Bottom) Overview of the RNA biomarker studies identified in the literature search. The seventeen studies identified were classified by the following attributes: performance metric, RNA type, tissue sampled, and species.

The search resulted in 17 manuscripts that have employed transcriptome-based methods to identify biomarkers for AUD or alcohol exposure (Figure 2). All 17 studies were designed to identify diagnostic biomarkers as the populations compared were AUD versus non-AUD, ethanol drinkers versus non-drinkers, or similar. It was evident that many of the studies assessed the effects of alcohol on the transcriptome in peripheral tissue without providing a metric of biomarker performance. Of the seventeen studies, only five included a measure of biomarker performance (Table 2). Of these five studies, four were in humans and one in mouse. Two used serum (one of these used serum exosomes), two used saliva, and one used whole blood. Three measured microRNAs, one circRNAs, and one mRNA. Overall, the ability of the peripheral transcriptional signatures to distinguish between AUD and non-AUD (or a similar alcohol-related phenotype, depending on the study) was good (AUC > 0.7) (Table 2).

**TABLE 2:** Alcohol transcriptome biomarker studies.

References	Species	Sample	RNA type	Population	Biomarker type	Biomarker performance			Repository accession
Liu et al., 2021	Human	Serum exosomes	Circular RNA	^●^ 6 AUD subjects. ^●^ 6 Healthy Controls. ^●^ 5 males, 1 female per group.	Diagnostic	AUC = 0.874 For circ_0004771. No model for circRNA combinations			NA
Ten Berg et al., 2018	Human	Serum	microRNA	^●^ 16 healthy participants (5 F, 11 M) before and after recreational ethanol consumption at a social event.	Diagnostic	AUC > 0.8: miR-375, miR-6879-3p and miR-4739 0.7 < AUC < 0.8: 148 other microRNAs species No model for miRNA combinations			NA
Rosato et al., 2019	Human	Saliva	microRNA	^●^ 60 AUD patients (28 AA: 16 F, 12 M, 32 EA: 16 F, 16 M). ^●^ 60 Control Subjects (28 AA: 16 F, 12 M, 32 EA: 16 F, 16 M).	Diagnostic	RF 80/20 train/test split	AA	EA	NA
						Top 10 miRNAs	73%	75.4%	
						Top 5 miRNAs	79%	72.2%	
						Top 3 miRNAs	76.4%	64.6%	
Mead et al., 2021	Human	Saliva	microRNA	^●^ 22 Alcohol abusers. ^●^ 15 non-abusing controls.	Diagnostic	AUC = 0.767 38 microRNA panel			NA
Ferguson et al., 2022	Mouse (C57BL/6J)	Whole blood	mRNA	^●^ 20 Chronic intermittent ethanol + 2BC voluntary EtOH test. ^●^ 20 Air-exposed + 2BC voluntary EtOH test. ^●^ 10 males and 10 females per group.	Diagnostic		F	M	GEO: GSE176122
						LR	0.901 (84.2%)	0.759 (66.7%)	
						RF	0.792 (77.8%)	0.582 (61.1%)	
						PLSDA	0.808 (78.9%)	0.805 (77.8%)	
						Values are AUC (accuracy)			

F, females; M, males; LR, logistic regression; PLSDA, partial least squares discriminant analysis; RF, random forest.

Twelve studies did not measure biomarker performance. Although these studies did not meet the inclusion criteria, they provide a basis for RNA biomarkers for AUD and so are tabulated separately (Table 3). Five were in humans, two included human and rat subjects, one was in mice, and four were in monkeys. Five studies assayed whole blood, two B cell-derived lymphoblastoid cell lines (LCLs), three PBMCs, one serum, and one plasma extracellular vesicles. Two studies measured microRNAs, three measured both microRNAs and mRNAs, one circRNA, and six measured mRNAs.

**TABLE 3:** Alcohol transcriptome studies in peripheral tissues (without biomarker performance evaluation).

References	Species	Sample	RNA type	Population	Repository accession
Gong et al., 2022	Mouse (C57BL/6J)	Whole blood	circRNA	^●^ 3 Chronic intermittent ethanol + 2BC voluntary EtOH test. ^●^ 3 Air-exposed + 2BC voluntary EtOH test. ^●^ All adult males.	SRA: PRJNA666616
Kupfer et al., 2013	Human	Whole blood	mRNA	^●^ 6 male subjects. Time course following OJ/vodka drink: baseline = BAC1, 0.04% = BAC2, 0.08% = BAC3, 0.04% = BAC4, and 0.02% = BAC5. ^●^ 5 male subjects. Time course following OJ (controls): T1 prior to drinking OJ, T2 at 90 min; T3 at 2 h, 49 min; T4 at 5 h, 8 min; and T5 at 7 h, 8 min.	GEO: GSE20489
Beech et al., 2012	Human	Whole blood	mRNA	^●^ 10 Abstinent alcohol dependent subjects (60% male, 50% Caucasian). ^●^ 13 heavy drinkers (77% male, 85% Caucasian). ^●^ 17 moderate drinkers (76% male, 82% Caucasian).	NA
Beech et al., 2014	Human	Whole blood	mRNA	^●^ 11 heavy drinkers (73% male, 91% Caucasian). ^●^ 11 moderate drinkers (73% male, 73% Caucasian). ^●^ Non-smoking, social drinking subjects. ^●^ Time course following exposure to three types of personalized imagery: neutral, stressful (but not alcohol-related), and alcohol-related cues (baseline, immediately after, and 1 h after stimulus presentation).	GEO: GSE59206
Hicks et al., 2012	Human; Rat (Long Evans)	Whole blood	mRNA	**Human** ^●^ 50 AUD subjects (18 F, 32 M). ^●^ 15 non-drinking control subjects (9 F, 6 M). **Rat** ^●^ 10 rats (5 M, 5F) on liquid ethanol diet for three consecutive days each week, followed by 4 days of ad lib solid rat chow pellets. ^●^ 10 pair-fed control rats (5M, 5F).	NA
McClintick et al., 2014	Human	LCLs	mRNA	^●^ 21 AUD (8 F, 12 M). ^●^ 21 drinking controls (9 F, 11 M). ^●^ Subjects from the Collaborative Study on the Genetics of Alcoholism (COGA). ^●^ 48 h exposure to 75 mM ethanol.	GEO: GSE52553
McClintick et al., 2019	Human	LCLs	mRNA	^●^ Same as McClintick et al. (2014) except 20 per group (two samples removed for technical reasons).	GEO: GSE126329
Barr et al., 2016	Rhesus macaque	PBMCs	mRNA and microRNA	^●^ 4 male heavy drinkers. ^●^ 4 male moderate drinkers. ^●^ 4 male controls. ^●^ 12 months of open access (22 h/day) to 4% EtOH in water solution. ^●^ Day 7 following vaccination with MVA.	mRNA: SRA: SRP064253 microRNA: SRA: SRP064540
Sureshchandra et al., 2016	Rhesus macaque	PBMCs	mRNA and microRNA	^●^ 5 female heavy drinkers. ^●^ 3 female controls. ^●^ 12 months of open access (22 h/day) to 4% EtOH in water solution.	SRA: SRP064925
Sureshchandra et al., 2019	Rhesus macaque	PBMCs	mRNA and microRNA	^●^ 4 male heavy drinkers. ^●^ 8 male moderate drinkers. ^●^ 3 male controls. ^●^ 12 months of open access (22 h/day) to 4% EtOH in water solution. ^●^ Also applied LPS to PBMCs (*ex vivo*).	SRA: PRJNA523863
Lewis et al., 2022	Rhesus macaque	Plasma extracellular vesicles	microRNA	^●^ 25 heavy drinkers. ^●^ 33 moderate drinkers. ^●^ 22 controls. ^●^ 12 months of open access (22 h/day) to 4% EtOH in water solution. ^●^ 17 females and 71 males across eight cohorts.	SRA: PRJNA769716
Ignacio et al., 2015	Human; Rat (Long Evans)	Serum	microRNA	**Human** ^●^ 20 AUD (10 F, 10 M). ^●^ 10 non-drinking controls (5 F, 5 M). **Rat** Daily drinking ^●^ 11 rats (6 male, 5 female) on liquid ethanol diet for 3 weeks. ^●^ 11 (6 male, 5 female) pair-fed (PF) controls. Intermittent access ^●^ 8 rats (4 male, 4 female) on intermittent liquid ethanol diet for 3 days each week, followed by 4 days of solid rat chow. ^●^ 8 (4 male, 4 female) PF controls.	GEO: GSE71579

### mRNA

Ten studies measured mRNA levels in peripheral tissues (Beech et al., 2012, 2014; Hicks et al., 2012; Kupfer et al., 2013; McClintick et al., 2014, 2019; Barr et al., 2016; Sureshchandra et al., 2016, 2019; Ferguson et al., 2022). Only one of these studies included a measure of biomarker performance (Ferguson et al., 2022). In that study, the authors conducted a multi-tissue analysis of brain (hypothalamus, prefrontal cortex, and amygdala) and blood gene expression after chronic intermittent ethanol exposure (CIE) in C57BL/6J mice (Ferguson et al., 2022). The ability of blood expression patterns to predict the treatment status of the mice (ethanol exposed or air exposed control) was assessed using three different ML classification techniques (LR with elastic net regularization, RF, and PLSDA). These were highly accurate (maximum AUC: 90.1%), suggesting that gene expression profiles from peripheral blood samples contain a biological signature of chronic ethanol exposure that can discriminate between CIE and Air subjects. Additionally, the within-subjects design enabled the comparison of blood and brain transcript levels in total and in responses to CIE. Regardless of treatment status (air or ethanol), there was a high degree of preservation between blood and brain transcript levels [rho range: (0.50, 0.67)]. There was small overlap between DEGs after CIE, and considerable overlap of gene networks perturbed after CIE related to cell-cell signaling (e.g., GABA and glutamate receptor signaling, Endocannabinoid signaling), immune responses (e.g., Role of JAK1, JAK2, and TYK2 in Interferon Signaling, B Cell Receptor Signaling, NFKB Signaling), and protein processing/mitochondrial functioning (e.g., Protein Ubiquitination Pathway, Oxidative Phosphorylation, EIF2 Signaling). This direct blood and brain comparison suggests that some ethanol-responses in the transcriptome are shared between blood and brain.

Another study linked peripheral blood mRNA levels with alcohol’s effects in brain (Hicks et al., 2012). This study took a targeted approach by analyzing genes related to p53 signaling, cell proliferation, apoptosis, and DNA repair in blood samples from a binge drinking rat model and human AUD and control subjects (Hicks et al., 2012). These specific genes were selected based on the group’s prior microarray study demonstrating that alcohol alters genes in these pathways when applied to cultured mouse NS-5 neural stem cells (NSCs) (Hicks et al., 2010; Hicks and Miller, 2011). Because blood leukocytes and NSCs are both highly proliferative cell populations, they hypothesized that similar expression changes would be observed following ethanol exposure. Blood gene expression for genes in these pathways were assessed after chronic ethanol in rats and in AUD subjects (Hicks et al., 2012). The rat binge drinking model showed significant expression changes in 190 out of the 350 genes assayed, and 40 of these were also differentially expressed in the mouse NSC study following ethanol exposure. The authors evaluated 34 of these genes in human AUD and control subjects and found that 7 of these genes (*HUS1, TP35, MYC, MUTYH, CDK4, ERCC1, MCM5*) were decreased in expression in human AUD blood compared with control. Blood expression levels of two genes (*ERCC1* and *MCM5*) showed a highly significant correlation with AUD-induced decreases in the volume of the left parietal supramarginal gyrus and various neuropsychological measures. This study is not hypothesis-free and does not include an evaluation of the biomarker utility (ability to discriminate between AUD and non-AUD individuals). However, it demonstrated an ethanol-related gene expression signature in human AUD blood and correlated blood gene expression levels with alcohol effects in the CNS.

Immune response is a prominent theme in peripheral mRNA transcriptomes after alcohol exposure, which is consistent with what is seen in brain (Mayfield et al., 2013; Crews et al., 2017; Erickson et al., 2019). In addition to the immune-related gene networks that were found to be similarly perturbed in the blood and brain of mice after chronic ethanol exposure as noted above (Ferguson et al., 2022), other peripheral transcriptome datasets highlight a similar finding in human blood. Beech et al. (2012) compared whole blood gene expression in abstinent alcohol dependent subjects (AUD), heavy drinkers, and moderate drinkers. 436 genes were differentially expressed among the three groups of subjects (FDR < 5%). 291 genes differed between AUD and moderate drinkers, 240 differed between AUD and heavy drinkers, but only 6 differed between heavy and moderate drinkers. Pathway analysis identified a common set of changes involving genes related to regulation of immune response by cytokines, T-cell receptors, and the JAK-STAT signaling pathway. These results suggest the transition from heavy alcohol use to dependence is accompanied by changes in the expression of genes involved in regulation of the immune response in whole blood.

To determine the relevance of these changes to stress and alcohol seeking behavior, Beech et al. (2014) performed a time course analysis of whole blood gene expression patterns in moderate and heavy drinkers after the presentation of neutral, stressful (but not alcohol-related), and alcohol-related cues. Gene expression was measured at three time points: baseline, immediately after, and 1 h after stimulus presentation. An “alcohol taste test” followed stimulus presentation in each condition. Subjects were allowed to drink up to 750 cc of beer and the amount of beer consumed was recorded. A repeated measures ANOVA revealed that 79 genes were changed by >1.3-fold in the heavy drinking group 1 h following exposure to the stress stimulus (FDR < 5%). No genes were identified as changed in either group immediately after cue presentation or following exposure to neutral or alcohol-related imagery. Pathway analysis suggested that many of these genes were related to translation and cell cycle regulation, and three of these genes (*RPL9, RPS3A*, and *RPS17*) form part of the transactivation responsive (TAR)-RNA-binding protein (TRBP)-associated complex and are positively regulated by miR-10a and miR-21. Expression of both miR-10a and miR-21 was measured by qPCR and found to be upregulated following the stress cue in heavy drinking (but not the moderate drinking) subjects. Expression levels of both microRNAs were correlated with amount of beer consumed in heavy drinking (but not moderate drinking) subjects (miR-10a, *R*^2^ = 0.59, miR-21, *R*^2^ = 0.57). Together these data that gene expression changes can be detected in response to psychological stress within an hour after cue presentation. Furthermore, expression of miR-10a, miR-21, and several of their target genes is regulated by psychological stress in heavy drinking subjects and correlated with stress-induced drinking in a laboratory setting.

Immune-related genes were also affected in blood after acute ethanol exposure. Kupfer et al. (2013) performed a time course analysis of acute ethanol on blood gene expression in healthy subjects as part of the civil aviation safety program to define the adverse effects of ethanol on flying performance. Subjects drank an orange juice and vodka mixture calculated to achieve a blood alcohol concentration of 0.08% wt/vol. Blood gene expression was profiled using microarrays at five time points: baseline, BAC = 0.04%, BAC = 0.08%, BAC = 0.04% during recovery, and BAC = 0.02%. Between 63 and 452 genes were differentially expressed between at least two time points depending on which method was used to identify DEGs. To cluster probe sets by temporal expression pattern, K-means clustering was performed on the 203 genes of interest (chosen based on arbitrary significance cutoff limits). All but four of the 203 genes fell into one of seven clusters. The genes in each cluster were enriched with genes related to similar biological processes, including hematological and immune functions (especially innate immunity), central metabolism, protein synthesis and modification, NF-κB signaling, inflammation, p38, MAPK, and small molecule metabolism. In addition to supporting immune effects of alcohol in the periphery, this study established that gene expression changes related to imbibed ethanol could be detected in blood within 90 min after ethanol intake.

McClintick and colleagues studied the effects of 24-h (McClintick et al., 2014) or 48-h (McClintick et al., 2019) exposure to 75 mM ethanol in LCLs from AUD and control subjects who were carefully diagnosed as part of the Collaborative Study on the Genetics of Alcoholism (COGA) Begleiter et al. (1995). LCLs are human cell lines derived from B-cells infected *in vitro* with the Epstein–Barr virus (EBV), a process that makes them immortalized. The group posited that LCLs are a useful model to study the effects of ethanol under controlled conditions and evaluate preexisting differences between those with and without AUD because (1) neuroimmune genes and pathways have been associated with AUD/ethanol in the brain, (2) many of the genes expressed in brain are also expressed in LCLs, and (3) LCLs are accessible. These studies showed that many genes were affected by ethanol exposure in LCLs. This included an immune response seen strongly after 24 h of exposure, that decreased in intensity after 48 h of exposure, potentially due to a reversal of interferon signaling. The genes that were increased in LCLs after ethanol exposure were enriched with genes related to pro-inflammatory pathways (e.g., IL6, dendritic cell maturation, TNF, and NFκB). The genes that were decreased in LCLs after ethanol exposure were enriched with genes related to the anti-inflammatory IL10 pathway. There was limited power to detect differences between untreated LCLs from AUD and controls. Combining the data between the two studies on unexposed cells identified 465 genes (nominal *p* < 0.05) with differences ≥1.2-fold. Three of these were identified as either genome-wide or nominally significant by GWAS and were affected by ethanol with a direction opposite those for AUD versus controls: *MREG* (melanoregulin), *CASZ1* (castor zinc finger 1), and *ST3GAL1* (ST3 beta-galactoside alpha-2,3-sialyltransferase 1). Sixty-seven pathways were enriched in the genes that differed at baseline between AUD and control LCLs. One of the most significantly enriched pathways was cholesterol biosynthesis (lower expression of *CYP51A1, HMGCR*, and *IDI1* in AUD LCLs relative to controls). Ingenuity Pathway Analysis software was used to predict what transcriptional regulators (e.g., transcription factors, microRNAs, kinases) are likely to produce the observed gene expression differences between AUD and control LCLs. This “upstream analysis” predicted higher activity of TLRs, LPS, TNF, interleukins, interferons, and TP53 [consistent with Hicks et al. (2012)].

Three studies have performed a transcriptome analysis using peripheral blood mononuclear cells (PBMCs) from rhesus macaque monkeys after chronic voluntary ethanol intake (Barr et al., 2016; Sureshchandra et al., 2016, 2019). Although these studies did not test the ability of these transcriptional changes to serve as distinguishing biomarkers (likely because of the low sample sizes), PCA clearly distinguished the transcriptional profiles of ethanol naïve and ethanol-consuming animals, with less separation between moderate and heavy ethanol consumers than between ethanol consumers and controls (Sureshchandra et al., 2016, 2019). This suggests that ethanol consumption is a main driver of PBMC gene expression variability and that the PBMC transcriptome would be useful in distinguishing ethanol-consuming and ethanol-naïve monkeys.

Consistent with the PCA results, there is a significant overlap between gene expression changes induced by moderate and heavy chronic alcohol drinking in macaques which is what was observed for human moderate and heavy drinkers as well (Beech et al., 2012). Transcriptional changes with heavy alcohol consumption were enriched with genes involved in cell-cell signaling, wound healing, coagulation, and immune system processes (Barr et al., 2016; Sureshchandra et al., 2019). Blood coagulation, immune signaling and wound healing pathways were exclusively detected with chronic heavy drinking (and not with moderate alcohol) (Sureshchandra et al., 2019). Barr et al. (2016), found that chronic heavy alcohol consumption increased the expression of genes associated with cancer and reduced the expression of immune genes involved in response to infection and wound healing, while chronic moderate alcohol consumption had the opposite effect (reduced the expression of genes involved in cancer and increased the expression of genes involved in immune response). This mirrors observations that AUD increases susceptibility to some viral and bacterial infections, whereas moderate alcohol consumption decreases the incidence of colds and improves immune responses to some pathogens (Messaoudi et al., 2013). Collectively, these findings indicate that the effects of ethanol on peripheral immune transcriptional signaling could be dose dependent, although there is strong overlap between the transcriptional signatures of moderate and heavy alcohol consumption in both monkeys (Barr et al., 2016; Sureshchandra et al., 2019) and humans (Beech et al., 2012).

Alcohol’s effects on immune signaling in blood is consistent with the fact that most mRNA in whole blood is from PBMCs, and PBMCs are responsible for initiating immune responses. One question is whether the differences in expression of immune signaling genes is caused by differences in immune cell numbers, differences in immune cell activity, or a combination of the two. Effects of alcohol on circulating immune cell counts have been inconsistent. The relative abundance of neutrophils was found to increase significantly in the blood of mice following CIE (Frank et al., 2020), but no changes in frequencies of circulating white blood cells were found between the ethanol consuming and control monkeys with chronic ethanol intake despite robust changes in gene expression, suggesting that alcohol is regulating the gene expression within immune cells in non-human primates (Sureshchandra et al., 2016, 2019). Another drug of abuse, amphetamine, decreased lymphocytes and increased monocytes in Sprague–Dawley rats, but it is unlikely that the change in cell frequencies explain the much greater fold changes observed in the mRNAs (e.g., almost a 6-fold increase for *Cd14*) (Bowyer et al., 2015).

In addition to immune signaling pathways, alcohol affected genes belonging to other major biological categories in peripheral tissues consistently across studies. Other broad categories include cell growth and death, translation, mitochondrial dysfunction/stress responses, and cell-cell signaling (among others). The cell signaling category contained pathways that are most well-studied in the central nervous system (CNS), e.g., GABA and glutamate signaling. Blood cells express ∼80% of genes that are expressed in brain including receptors and enzymes that are required for processing the main excitatory and inhibitory neurotransmitters of the CNS (glutamate and GABA, respectively), and other neurotransmitter and neuropeptide systems (Gladkevich et al., 2004; Liew et al., 2006; Sedaghati et al., 2010; Tylee et al., 2013; Bhandage et al., 2015; Luykx et al., 2016; Shao et al., 2021). In fact, cell-cell signaling was one of the most highly affected biological categories by alcohol in some studies (Kupfer et al., 2013; Sureshchandra et al., 2016; Ferguson et al., 2022). Genes in the cell-cell signaling category included various glutamatergic signaling genes (e.g., Grm5, Homer1), GABA signaling genes (e.g., Gabbr1, Gabra2, Gabrb1, Slc6a1), potassium channels (e.g., Kcnq2, Kcnn4), calcium channels (Cacna1a, Cacna1b), amongst others. Further study is required to determine whether alcohol affects these neurotransmitter systems in the periphery in the same way as in the CNS and whether the peripheral state of these “neurotransmitter systems” is reflective of their state in brain tissues. The studies discussed in this Review demonstrate that components of these neurotransmitter systems are detected in peripheral blood and are responsive to ethanol consumption. These findings support the use of systems level approaches that incorporate information derived from peripheral tissues into AUD research and in neuroscience more broadly.

### Non-coding RNAs

Increasing evidence suggests that non-coding RNAs (ncRNAs) play a role in psychiatric disorders including AUD (Mayfield, 2017). The effects of alcohol on ncRNAs have been explored in both brain (Bohnsack et al., 2019; Drake et al., 2020) and peripheral tissues (Ignacio et al., 2015; Momen-Heravi et al., 2015; Barr et al., 2016; Sureshchandra et al., 2016, 2019; Ten Berg et al., 2018; Rosato et al., 2019; Liu et al., 2021; Mead et al., 2021; Gong et al., 2022; Lewis et al., 2022). The studies identified in the literature search analyzed two types of ncRNAs in peripheral tissues: microRNAs and circRNAs. There is a paucity of information on other regulatory ncRNAs such as lncRNAs and piRNAs in peripheral tissues, so these represent unexplored areas of research for future study.

#### microRNAs

Eight studies measured microRNA levels in peripheral tissues (Ignacio et al., 2015; Barr et al., 2016; Sureshchandra et al., 2016, 2019; Ten Berg et al., 2018; Rosato et al., 2019; Mead et al., 2021; Lewis et al., 2022; Tables 2, 3). MicroRNAs are short RNAs (about 20–24 nucleotides long) that regulate mRNA expression at the post-transcriptional level by repressing the translation or cleaving the transcript (Lagos-Quintana et al., 2001; Bartel, 2004). MicroRNAs are attractive AUD biomarker candidates for several reasons: (1) they can be actively or passively excreted into fluids including saliva, tears, CSF, and blood (usually circulating in extracellular vesicles such as exosomes), (2) they can retain organ specificity (for certain microRNA species), (3) miRNAs in exosomes have been shown to cross the BBB and facilitate peripheral-central crosstalk, (4) they are relatively stable in blood, saliva, and urine (reviewed in Mitchell et al., 2008; Cortez et al., 2011; Hayes et al., 2014; Schwarzenbach et al., 2014; Glinge et al., 2017; van den Berg et al., 2020).

Three studies assessed the diagnostic discrimination of peripheral microRNA profiles for recreational alcohol drinking (Ten Berg et al., 2018), AUD (Rosato et al., 2019), and alcohol abuse (Mead et al., 2021; Table 2). The first study demonstrated that circulating microRNAs are dynamic and highly predictive of drinking even a modest amount of alcohol (Ten Berg et al., 2018). This study determined the effect of attending a social event and consuming alcohol on circulating microRNAs in 16 healthy adults by applying small RNA sequencing to serum samples collected up to 48 h before and 3–5 h after recreational exposure to alcohol. The median ethanol concentration was 89 mg/dL, which is only slightly above the alcohol limit for driving (80 mg/dL). Around a fifth of the microRNAs detected in human serum (265/1370 microRNAs) increased by more than 2-fold after attending a social event with recreational alcohol ingestion. There was only 1 microRNA (miR-185-5p) that was decreased following alcohol exposure (FDR < 5%, fold decrease 2 or more). By contrast, conventional hematology and biochemistry parameters (ALT, GGT, full blood count, creatinine, bilirubin, urea and electrolytes, alkaline phosphatase) were unaffected. Three microRNA species separated post-alcohol exposure from pre-exposure with AUC values > 0.8 (miR-375, miR-6879-3p, and miR-4739), and 148 microRNA species had AUC values between 0.7 and 0.8. The study did not evaluate the combination of multiple microRNAs on predictive performance.

Rosato et al. (2019) profiled miRNA transcriptomes in the saliva of 120 AUD and healthy control subjects (56 African–American AAs and 64 European–American EAs). The group analyzed expression differences in 399 microRNAs that were very highly expressed (>100 CPM) in at least half the samples (although 2,588 miRNAs were detected in the saliva). Seven microRNAs were differentially expressed in African–Americans (miR-451a, miR-10a-5p, miR-100-5p, miR-3613-5p, miR-7704, miR-1290, and miR-4488) and five in European–Americans (miR-126-3p, miR-10a-5p, miR-1290, miR-4488, and miR-1273h-5p) (*p* < 0.05 and fold-change > 2). Three miRNAs (miR-4488, miR-1290, and miR-10a-5p) showed similar fold changes and the same direction of expression changes in both AA and EA AUD subjects. miR-10a-5p is especially noteworthy as it has also been independently validated as being upregulated in the saliva of an alcohol abusing population (Mead et al., 2021) and in the blood of heavy drinkers (but not moderate drinkers) after acute psychological stress and is correlated with stress-induced drinking in a laboratory setting as described in the Section “mRNA” above (Beech et al., 2014). Enrichment analysis was conducted on the mRNAs potentially targeted by these three differentially expressed miRNAs. The targets of miR-10a-5p were related to DNA binding, miR-1290 with alternative splicing, and miR-4488 with calcium-dependent cell–cell adhesion. The RF ML algorithm was used to determine whether the 399 highly expressed miRNAs could predict AUD status. When the top five miRNAs (ranked by Gini index of their importance to AUD prediction) were included in the RF model, the AUD prediction accuracy was 79.1 and 72.2% in AAs and EAs, respectively (Table 2). Inclusion of the top ten miRNAs (ranked by Gini index or their importance to AUD prediction) in the RF prediction analyses decreased the AUD prediction accuracy to 73.6% in AAs but slightly increased it to 75.4% in EAs. If the top three miRNAs (ranked by Gini index or their importance to AUD prediction) were included in RF prediction analyses, the AUD prediction accuracy was decreased in AAs (76.4%) and to a greater extent in EAs (64.6%). If the differentially expressed miRNAs (*p* < 0.05 and logFC > 1) were included in the prediction analysis, the prediction accuracy was the lowest (63.6% in AAs and 56.2% in EAs), suggesting that feature selection using ML could be more powerful in identifying discriminative biomarkers for predicting AUD status than using differential expression, although the top miRNAs according to the Gini importance measure were also differentially expressed in AUD subjects (e.g., miR-4488).

In addition to AUD, there is also evidence that microRNA profiles from saliva might be useful in distinguishing heavy alcohol drinkers from sporadic alcohol users (Mead et al., 2021). In this study, saliva was collected from 37 patients at least 48 h after being admitted to a primary healthcare center for an underlying medical condition requiring hospitalization (e.g., pneumonia, acute kidney injury, chronic kidney disease secondary to hypertension, secondary diabetes mellitus, chest pain, ischemic heart disease, congestive heart failure, or cardiac arrhythmia). MicroRNA was profiled from saliva samples using microRNA microarrays (v3.0, Applied Biosystems, Foster City, CA, United States). Comparison of microRNA expression levels between alcohol abusers and controls revealed 38 significantly (*p* < 0.05) changed microRNAs: 15 downregulated microRNAs (miR-132-3p, miR-136-5p, miR-146a-5p, miR-146b-3p, miR-194-5p, miR-20b-5p, miR-26a-5p, miR-26b-5p, miR-422a, miR-487a, miR-590-5p, miR-618-3p, miR-628-5p, miR-652-3p, and miR-9-5p), 10 upregulated microRNAs (miR-184, miR-20a-5p, miR-223-5p, miR-27a-3p, miR-30b-5p, miR-34a-5p, miR-449a, miR-483-5p, miR-500-3p, and miR-744-5p), and 13 *de novo* microRNAs (let-7e-5p, miR-1, miR-10a-5p, miR-10-3p, miR-1249, miR-182-5p, miR-183-5p, miR-18b-5p, miR-196b-5p, miR-221-5p, miR-490-3p, miR-548-5p, and miR-450b-5p). The top pathways enriched in the potential targets of the microRNAs differentially expressed in patients chronically abusing alcohol were: Adherens junction [consistent with what was seen in Rosato et al. (2019)], Endocytosis, Wnt signaling pathway, MAPK signaling pathway, and ErbB signaling pathway. A plot of the first three principal components showed that most of alcohol abusing patients and controls separated into different clusters with minimal overlap based on saliva miRNA expression profiles. Consistently, the panel of 38 differentially expressed microRNAs was able to distinguish between the alcohol abusing patients and controls very well with an AUC of 0.7668. However, there was not enough information provided in the manuscript to determine whether this result suffers from data leakage, a common pitfall in predictive modeling (Luo et al., 2016). This occurs when information used to select features (i.e., microRNAs) for the classifier is also used to test the classifier. In other words, if the top differentially expressed microRNAs were selected using the entire dataset, and then the entire dataset was used to test how well the DEGs can distinguish between the groups, the prediction accuracy will be inflated (likely much higher than it would perform in an independent dataset or if cross validation was used). Another important finding from this study was that the two main cell types in saliva are squamous epithelial buccal mucosa cells and leukocytes as indicated by morphological and flow cytometry data. The fact that leukocytes can infiltrate the oral cavity may be especially pertinent to AUD in which peripheral and central immune processes are greatly affected as already noted.

Five additional studies measured ethanol’s effects on microRNA expression in accessible tissues but did not include any metric of biomarker utility (Ignacio et al., 2015; Barr et al., 2016; Sureshchandra et al., 2016, 2019; Lewis et al., 2022). However, one of these studies demonstrated a correlation between serum miRNAs and alcohol-related structural and functional CNS damage in human subjects (Ignacio et al., 2015). This study identified 13 upregulated microRNAs (mir-96, mir-320b-1, mir-1976, mir-24-1, mir-30a, miR-96-5p, mir-127, mir-136, mir-320b-2, mir-421, mir-671, mir-3615, mir-3676) and 3 downregulated microRNAs (mir-92b, miR-301a-3p, miR-660-5p) in AUD serum samples compared with control. All but four of these differentially expressed microRNAs were correlated with either GGT levels, drinking amounts, or neuroimaging variables that were significantly different in AUD subjects. The finding that most of the microRNAs were upregulated is consistent with alcohol’s effects on microRNAs reported in serum (Ten Berg et al., 2018) and also in post-mortem prefrontal cortex samples from AUD subjects (Lewohl et al., 2011), but not saliva where there were similar numbers of up- and downregulated microRNAs (Rosato et al., 2019; Mead et al., 2021) or monkey PBMCs where there were either more downregulated microRNAs (Sureshchandra et al., 2016, 2019; Lewis et al., 2022) or roughly equal numbers of up- and downregulated microRNAs (Barr et al., 2016; Lewis et al., 2022). This study also analyzed serum miRNA levels in a rat drinking model and found that 3 of the top 5 molecular functions represented in the altered miRNAs overlapped across species: (1) cellular development, (2) cell growth and proliferation, and (3) cell death and survival. These results provide evidence that alcohol induces global miRNA expression changes in serum that can reflect CNS alcohol-related changes.

The macaque studies discussed in the Section “mRNA” above also analyzed microRNA profiles in the same PBMC samples (Barr et al., 2016; Sureshchandra et al., 2016, 2019). Barr et al. (2016) compared the miRNA expression profiles of the PBMCs isolated from male controls, moderate, and heavy drinkers on day 7 after a Modified Vaccinia Ankara vaccination. The largest differences in miRNA expression were observed between controls and heavy drinkers, and only a few miRNAs were differentially expressed between controls and moderate drinkers, which mirrored the mRNA (Barr et al., 2016). Of the 79 differentially expressed miRNAs between controls and heavy drinkers, 37 were upregulated and 42 were downregulated (at a fold change ≥ 2 and a FDR < 5%) (Barr et al., 2016). MicroRNAs usually repress transcription levels of their target mRNAs, so if a microRNA is downregulated then the expression of its target mRNAs would be expected to increase, and if a microRNA is upregulated then the expression of its target mRNAs would be expected to decrease. The analysis of PBMC mRNA levels from the same subjects revealed that ∼40% of the differentially expressed microRNAs had mRNA targets changed in the opposite direction (Barr et al., 2016).

Another study profiled microRNAs in PBMCs from male heavy drinking, moderate drinking, and control monkeys (without any vaccination in contrast to the Barr et al. study). Chronic heavy alcohol drinking resulted in the downregulation of nine microRNAs (miR-23a, miR-204, miR-211, miR-668, miR-1226, miR-154, miR-9-1, miR-9-2, miR-9-3) and upregulation of 2 microRNAs (miR-27b, miR-203) (Sureshchandra et al., 2019). About 10% of the mRNAs that were differentially expressed with heavy alcohol drinking were validated targets of these miRNAs. These mRNAs play roles in regulation of body fluids, coagulation, and wound healing (Sureshchandra et al., 2019). Moderate drinking in male monkeys was associated with 24 differentially expressed miRNAs, which again were also mostly downregulated with drinking (19 of 24). Five were also downregulated with heavy drinking (miR-9-1, miR-9-2, miR-9-3, miR-668, miR-154) and 14 were only downregulated with moderate drinking (miR-150, miR-31, miR-221, miR-92b, miR-377, miR-181-a1, miR-181-a2, miR-380, miR-339, miR-484, miR-494, miR-542, miR-184, miR-497). Approximately 20% of the mRNAs affected by moderate drinking are validated gene targets of these microRNAs. Moderate drinking upregulated 3 microRNAs which were all different than those upregulated with heavy drinking (miR-196, miR-143, and miR-627). The fact that more microRNAs were altered with moderate drinking than heavy drinking is in contrast to other studies at both the mRNA and microRNA levels which found that heavy drinking had a greater effect on peripheral transcriptomes than moderate drinking. In female macaques, chronic heavy drinking resulted in a downregulation of 4 microRNAs in PBMCs: miR-23a (which was also downregulated in male PBMCs after heavy drinking), miR-27a, miR-24, miR-663 (Sureshchandra et al., 2016). Three of miR-27a target mRNAs were upregulated (*PAQR9, NR2F6*, and *GATA2*), two of miR-24 target mRNAs were upregulated (*NEFM, BNIP3L*), and two of miR-23a target mRNAs were upregulated (*CA2, PTP4A2*). This study did not use a small RNA library preparation so there was limited ability to detect microRNAs.

The final macaque microRNA study to be discussed was the largest cohort size (*N* = 80; 17 females and 71 males across eight cohorts) and analyzed plasma extracellular vesicles instead of PBMCs (Lewis et al., 2022). These samples were from the same animals used for two PBMC studies discussed in the previous paragraph (Sureshchandra et al., 2016, 2019). In female heavy drinking monkeys, differential expression analysis identified 8 upregulated microRNAs (mir-544, mir-3064, mir-208b, mir-371, mir-154, mir-124a, mir-4800, mir-889, mir-141) and 21 downregulated microRNAs (mir-147b, mir-1224, mir-650c, mir-650a, mir-548AN, mir-216a, mir-526A1, mir-6125, mir-212, mir-371, mir-890, mir-548F1, mir-488, mir-625, mir-523, mir-219, mir-517B, mir-1179, mir-521, mir-203, mir-205). For males there were 9 upregulated microRNAs (mir-544, mir-3064, mir-208b, mir-371, mir-154, mir-124a, mir-4800, mir-889, mir-141) and 6 downregulated microRNAs (mir-577, mir-580, mir-1224, mir-935, mir-6790, mir-1469). Only one upregulated (miR-154) and one downregulated (miR-1224) microRNAs were common between males and females. The predicted mRNA targets of the upregulated microRNAs were enriched with gene ontology terms associated with blood vessel development (e.g., miR-124a, 130b, 141 and 34c), response to reactive oxygen species (e.g., miR-124a, 433, 493, and 34c), myeloid leukocyte activation (e.g., miR-124a, 130b, 154, 208b, 544). The predicted mRNA targets of the downregulated microRNAs were enriched with gene ontology terms associated with signaling (e.g., “regulation of protein kinase activity”), cell cycle (e.g., “mitotic DNA damage checkpoint”), and tissue/epithelial homeostasis (e.g., “tissue homeostasis” and “response to hypoxia”). These data show that chronic ethanol consumption alters the expression of several extracellular vesicle miRNAs that can potentially serve as biomarkers of chronic ethanol consumption.

#### Circular RNAs

Circular RNAs (circRNAs) are a class of RNA species that lack 5′ N7-methylguanosine (m7G) caps and 3′ polyadenylated tails, and instead are single-stranded RNAs covalently closed in a circular formation. CircRNAs are usually produced through a process called pre-mRNA “back-splicing” of exons, introns, and intergenic regions (Liu and Chen, 2022). circRNAs play roles in multiple biological processes, such as modulation of transcription and translation (Liu and Chen, 2022), brain development and function (Veno et al., 2015; Piwecka et al., 2017), cell cycle control (Zheng et al., 2016), cancer onset and progression (Guarnerio et al., 2016), ribosome biogenesis (Holdt et al., 2016), and endothelial and vascular function (Shan et al., 2017). Like microRNAs, circRNAs are relatively stable with half-lives ranging from 18.8 to 23.7 h (compared with 4.0–7.4 h for their cognate linear RNAs) (Enuka et al., 2016) and are found both inside their cells of origin as well as in extracellular fluids, including blood and plasma, extracellular vesicles, saliva, and urine, which supports their use as biomarkers (reviewed in Liu and Chen, 2022).

Circular RNAs were sequenced in AUD and control samples from circulating serum exosomes (Liu et al., 2021). Differential expression analysis identified 254 differentially expressed circRNAs. Of these, 149 were upregulated and 105 were downregulated (*P* < 0.05). CircRNAs can act as microRNA sponges (Hansen et al., 2013). To determine potential effects on microRNAs, the authors analyzed the predicted target miRNAs of the differentially expressed circRNAs. The analysis showed that miR-4739, miR-1248-5p, miR-3685, and miR-6751-5p were regulated by a greater number of circRNAs than other miRNAs. miR-4739 was predicted to be targeted by 15 of the 254 differentially expressed circRNAs. This microRNA was also highly upregulated (3.47-fold) after recreational alcohol consumption in healthy young people (Ten Berg et al., 2018). In fact, it was one of the three miRNAs that could predict alcohol exposure with an AUC > 0.80. The expression of one of the top differentially expressed circRNAs, circ_0004771, was validated in an independent cohort of AUD patients’ serum exosomes by qRT-PCR and confirmed to be upregulated in AUD samples. Expression levels of exosomal hsa_circ_0004771 were able to distinguish between AUD and non-AUD samples with an AUC of 0.874. Moreover, the expression level of exosomal hsa_circ_0004771 was found to be highly correlated with AUD severity as measured by the Severity of Alcohol Dependence Questionnaire (SADQ) SADQ and the Alcohol Dependence Scale (ADS) (*r* = 0.8328 and 0.8411, respectively).

To explore whether circRNA could be a biomarker for chronic ethanol exposure, Gong et al. (2022) assayed circRNA in whole brain and blood samples from adult male C57BL/6J mice that had been exposed to either chronic intermittent ethanol (CIE) or air. A total of 399 circRNAs were differentially expressed in CIE mice relative to controls. Of these, 150 circRNAs were significantly upregulated and 249 were downregulated in brain tissue from the CIE groups compared with the control brain tissue. The most enriched KEGG pathways of the parental genes were GABAergic synapse, retrograde endocannabinoid (eCB) signaling, and morphine addiction. Among the DE circRNAs, qRT-PCR confirmed 14 upregulated and 13 downregulated circRNAs in brain. The authors measured these 27 circRNAs in blood samples from the same mice and found that 9 circRNAs were also upregulated in blood, and 10 circRNAs were also downregulated in blood samples. The fold changes were much higher in blood than brain (2.5–14-fold upregulated in brain versus 10–150-fold in blood). This study suggests that chronic ethanol exposure can affect circRNA expression in whole blood in a similar direction as brain, and that ethanol’s effects on these circRNAs are even stronger (i.e., of a greater magnitude) in blood than brain.

## Considerations, limitations, and future directions

### Biomarker research is bound by current nosology which is imperfect

The search for objective diagnostic molecular biomarkers is based on identifying the molecular differences between two groups. However, the groups are defined by “biased” diagnostic criteria (e.g., DSM, ICD, AUDIT, other self-reported assessments). This circular process is a major limitation for this research area. Illustrating this point, an epigenome wide association study revealed DNA methylation marks were associated with PETH (an objective measurement of alcohol consumption) but not to AUDIT-C scores (a self-reported measurement of alcohol consumption), despite there being a positive correlation between PETH and AUDIT-C (Liang et al., 2021). Frameworks incorporating dimensional approaches have been recommended to address this limitation by reconceptualizing the nosology of AUD on the basis of process and etiology, e.g., Addictions Neuroclinical Assessment (ANA) and Alcohol Addiction RDoC (AARDoC) (Litten et al., 2015; Kwako et al., 2016, 2018). This research field will progress in an iterative process between biomarker development, trials of novel interventions, mechanistic studies, and ongoing nosological refinement (Ewen et al., 2021).

### Biomarkers are not necessarily causal

Blood biomarkers may represent a combination of AUD-specific factors (factors that are mechanistically related to AUD) and non-specific factors (factors that are associated with AUD, but not mechanistically relevant). First and foremost, the biomarker must be sensitive and specific at its task whether that be diagnosis, prognosis, treatment response, or treatment prioritization. However, an ideal biomarker would not only be a “marker” but would also reflect the underlying pathophysiology (Heilig et al., 2016).

Two studies discussed in this review showed similar changes in brain and blood for some RNA expression levels with alcohol exposure (Ferguson et al., 2022; Gong et al., 2022). Other studies linked peripheral RNA levels with alcohol-related structural and functional CNS damage, various neuropsychological measures, and AUD severity (Hicks et al., 2012; Ignacio et al., 2015; Liu et al., 2021). This is consistent with other studies, for example, that have found that 85-90% of the most predictive molecular pathways identified in the brain are also top predictors in the blood for neurodegeneration (Iturria-Medina et al., 2020). Of course, blood and brain molecular measurements are not always concordant. Altered plasma levels of some miRNAs did not reflect the levels found in cerebellar tissue (Rossetto et al., 2019) or prefrontal cortical tissue (Xin et al., 2018) with ethanol exposure in rats, but these studies only looked at a small number of microRNAs and did not conduct a global survey of microRNA expression. It is possible that some microRNAs would have reflected brain levels. Co-expression networks were more conserved between brain and blood than individual genes for chronic ethanol exposure in mice (Ferguson et al., 2022). This is also supported by other studies that have shown moderate correlation between expression profiles in blood and brain for individual genes (Liew et al., 2006; Tylee et al., 2013), but preservation of ∼90% of gene networks between blood and prefrontal cortex tissue (Hess et al., 2016). This further supports the use of gene signatures rather than individual genes.

### Confounding role of similar or comorbid conditions and medications

The studies in this Review compare an alcohol dependent population to healthy controls with exclusion criteria often including other psychiatric illnesses or diseases. Some of the pathways identified in these studies that are disrupted in peripheral tissue with AUD have been linked with numerous other diseases (e.g., inflammatory/immune responses, extracellular matrix, cell cycle, mitochondrial dysfunction) (Crow et al., 2019). Moreover, some genes are likely to be differentially expressed in a wide range of conditions (Crow et al., 2019). Combined with the high rates of comorbidity and shared genetic mechanisms between AUD and some of these other disorders (Evangelou et al., 2019), identifying AUD-specific biomarkers represents a significant challenge. One investigation found that using single plasma analytes to identify individuals with Alzheimer’s disease could lead to the misdiagnosis of individuals with certain comorbidities (e.g., chronic kidney disease) (Mielke et al., 2022). Using multiple RNA biomarkers (a panel/signature) will be important for specificity, and high performance will likely require incorporating additional alcohol-related markers from other levels of analysis (e.g., protein/metabolic, epigenetic, clinical/demographic) with the RNA panel. Furthermore, future studies should incorporate other groups to determine the biomarkers’ ability to discriminate between similar and comorbid disorders.

Another confound for AUD biomarker research is the use of medications. The transcriptome can respond to medications, and if individuals with AUD are taking medications to treat their AUD (or other conditions) this could confound the RNA biomarker search. For example, some studies have found very little disorder-related genes independent of treatment for schizophrenia and bipolar disorder (Krebs et al., 2020; Sershen et al., 2021). It is important to understand what factors influence the levels of peripheral biomarkers for clinical use, and to establish appropriate reference ranges. For example, circulating miR-122 levels are increased about 2-fold with modest alcohol consumption in healthy individuals (McCrae et al., 2016; Ten Berg et al., 2018). Expression of miR122 is used as a biomarker of liver injury, which has a median fold increase around 100, so in this case alcohol consumption would not be a confounding factor, but it illustrates the point that reference ranges must be considered to have confidence that environmental factors are not going to produce false positive or negative results.

### Other types of biomarkers besides diagnostic biomarkers, and the need for longitudinal studies

All the studies discussed in this Review used RNA profiles from peripheral tissues as diagnostic biomarkers to detect ethanol exposure or AUD status. However, in addition to providing data-driven information to guide AUD diagnosis, peripheral RNA profiles could also be utilized as other types of biomarkers listed in Table 1. For example, RNA profiles could be used to identify individuals at high risk for developing AUD (risk biomarker), stratify the heterogeneous AUD patient population into subtypes (this would also be considered a diagnostic biomarker), select the optimal treatment (predictive biomarker) and monitor treatment efficacy (monitoring biomarker; response biomarker), or predict those most likely to recover from AUD or track AUD status (prognostic biomarker).

Some of these biomarkers are related to one another. For example, stratifying patients into subgroups could facilitate optimal treatment selection. There is some evidence that naltrexone is more effective for reward drinkers (Mann et al., 2018) and that acamprosate is more effective for relief drinkers (Roos et al., 2017). There have been some efforts to develop a questionnaire to identify reward, relief, and habit drinkers but there was group heterogeneity and the relief and habit groups were not as reliable upon retest as the reward group (Grodin et al., 2019). Future studies that combine the questionnaires with whole-genome unbiased molecular profiles could begin to narrow down a panel of biomarkers that might help to better distinguish these classes. If a molecular signature could be defined for reward and relief drinkers, this could help prioritize naltrexone or acamprosate for their treatment.

Developing these RNA biomarkers will require longitudinal studies, where samples are taken repeatedly before the onset of AUD and throughout AUD progression. Inevitably some participants in these studies would develop AUD (and some not), and some of those who develop an AUD will recover either with intervention or spontaneously (and some not). Data from these studies will be critical in developing other types of RNA biomarkers for AUD, which could arguably represent a greater healthcare need than diagnostic biomarkers because there are existing tools to diagnose AUD (albeit with limitations), but there are currently no ways to identify “at-risk” individuals, stratify the AUD patient population, select optimal treatments, or monitor treatment response.

### CMap approaches

Gene expression data can be used to identify pharmacological treatments for AUD through a process termed connectivity mapping (Lamb et al., 2006; Subramanian et al., 2017; Ferguson et al., 2018). The idea behind this approach is that pharmacological compounds that have opposing effects on gene expression as observed with AUD would be able to treat AUD. There has been some success in animal models of AUD and for other brain diseases using brain gene expression (Ferguson et al., 2017, 2018). It remains to be seen whether blood signatures can be used as an accessible transcriptome for connectivity mapping approaches for AUD, and future work will need to carefully evaluate this possibility. If this is the case, then connectivity mapping approaches could be dynamically applied for AUD. For example, it is plausible that treatment for AUD will change throughout the course of the disorder; that the treatments during acute withdrawal will be different than treatments during protracted withdrawal, and that treatments will not be necessary at all at some point. Blood and saliva can be sampled repeatedly which affords the opportunity to monitor AUD progress and treatment efficacy, and dynamically select optimal treatments.

### Single cell approaches

The studies in this Review used bulk sequencing techniques which are low cost, scalable, and can identify differential expression and co-expression between patients and controls. However, a key source of unwanted variability is cellular heterogeneity and unwanted variability can decrease statistical power to detect meaningful differences between groups of interest. Imagine a scenario in which ethanol strongly upregulates an RNA in one cell type, and strongly downregulates the same RNA in another cell type. In bulk sequencing, the cells are lysed and the RNAs in each cell are combined. This effectively averages ethanol’s effects across cell types, and RNAs that are affected strongly by ethanol (but in different directions depending on cell type) would be overlooked. Single-cell techniques use next-generation sequencing to analyze the genetic content of individual cells, providing valuable insights into ethanol’s effects on the functional characteristics of each cell type. Two challenges with single cell data are that it is difficult to generate cell type specific co-expression networks and cost is high which diminishes the clinical utility of single cell approaches. However, single cell data will be critical in understanding the mechanistic relevance of peripheral biomarkers to AUD which is a key part of the process in AUD biomarker development.

### Artificial intelligence

Artificial intelligence (AI) tools, like the ML classifiers described in the Section “Evaluating biomarker performance,” are increasingly being explored in medical and healthcare research, and there are currently 79 AI/ML based, FDA approved medical devices and algorithms^1^ (Benjamens et al., 2020). The word tool is used deliberately here to emphasize the point that AI cannot replace the judgment of a physician. Rather AI tools can provide probabilities and likelihoods that are part of a holistic picture that the physician can consider when making a clinical decision about a patient. This has been shown to be the case for ML tools developed to detect prostate cancer from MRI scans, which did not persuade physicians to change their diagnostic decision in the rare instance that an incorrect probability was assigned by the algorithm. But, overall the AI tool improved physicians’ diagnostic accuracy and reduced physicians’ variability (Sun et al., 2022).

Two studies in this Review used ML classifiers to predict AUD or chronic ethanol exposure from transcriptomic data (Rosato et al., 2019; Ferguson et al., 2022). Other types of data (e.g., EEG measures, SNPs, MRI data) have also been used to train ML classifiers to predict alcohol-related phenotypes. One study used structural MRI data collected from multiple sites as input into LR models to predict AUD status and achieved an AUC of 0.768 (Hahn et al., 2022). An important consideration in ML that is illustrated by this study is that of group imbalances. The data across separate sites were highly imbalanced (i.e., many sites contained only participants with AUD or only controls), so the ML classifiers appeared to distinguish participants with AUD but were actually learning site-related effects like scanner and demographic differences. These effects were mitigated, and the models made more generalizable, by the feature selection strategy the authors used (discussed below). Another study used EEG measures and alcohol-related SNPs to train SVM ML models to predict those at-risk for developing AUD (Kinreich et al., 2021). The group first used regularized LR to select features to use in the SVM models, and found that the combined EEG and SNP features model outperformed models based on only EEG features or only SNP features with an AUC of 0.84 for European American subjects and 0.90 for African American subjects (Kinreich et al., 2021). Another study used SVM models to predict alcohol exposure in rats based on MRI data. The authors took a similar two-step approach and used a RF algorithm to select the features with which to train the SVM models and found that 1 month of ethanol exposure was enough to imprint a highly specific signature of alcohol consumption so that the models could distinguish between ethanol-naïve and ethanol exposed samples with near perfect accuracy (Cosa et al., 2017). The study also showed that the models could predict which samples had been treated with naltrexone using MRI features with greater than 84% accuracy. LR models have also been used to predict acamprosate response from metabolomic data (Hinton et al., 2017). The models predicted acamprosate treatment response with a mean AUC of 0.647. The mean sensitivity in the test was 0.83 and the mean specificity in the test was 0.31, suggesting that the model performed well at predicting responders but not non-responders (i.e., many non-responders were predicted to respond) (Hinton et al., 2017). Taken together, these studies demonstrate that the AUD research field has begun to apply ML methods that can guide diagnosis and treatment response, and additionally might help in identifying biologically meaningful features of AUD. However, in addition to class imbalances, there are several other considerations when using ML approaches with transcriptomic and other omics datasets.

One of the biggest challenges when using ML approaches with gene expression data is known as the “curse of dimensionality,” which means that the number of observations is greater than the number of samples. Gene expression data sets (and other -omics data sets) are high dimensional, meaning that there are many observations (tens of thousands of gene expression levels are measured). One way to solve the curse of dimensionality is to increase the sample size. There is scarcity of research in training set size determination methodologies for ML, but it will depend on the complexity of the algorithm and the specific use case. The studies discussed in the above paragraph range in sample size from 20 to 2,034. When large sample sizes are not readily available, another solution is to reduce the number of features (i.e., genes) using feature selection techniques. This is advisable as many of the features in high dimensional datasets will be either irrelevant or redundant which will reduce the power of the ML algorithm to identify meaningful patterns (Plant and Barton, 2021). Some ML models have “built-in” feature selection, and we discussed these examples above. In regularized LR models some of the coefficients will go to zero so these features will be removed from the model. For the RF algorithm, some of the features will be assigned importance scores of zero and will not be included in the model. The cross-site MRI study discussed above used a genetic algorithm (GA) based feature selection search which repeatedly trained and evaluated a regularized LR classifier on random subsets of brain features (Hahn et al., 2022). In this way the features selected were derived from thousands of models, providing the chance for highly correlated features to achieve similar importance scores. Care must be taken to not build the models using features that were pre-selected from the complete dataset (Plant and Barton, 2021). Otherwise, this could result in “data leakage” and overestimate the accuracy of the model [discussed for the study by Mead et al. (2021) in the Section “microRNA” above].

In summary, ML models learn a function from training data that consist of pairs of input objects (e.g., gene expression signatures) and assigned labels (e.g., AUD or non-AUD) and the model’s performance is then evaluated on an independent testing dataset. The training data set is important both in size and nature. The more examples in a training set and the more representative it is of the patient population, the better the model will be in terms of accuracy and generalizability. To increase the ability of ML algorithms to build meaningful (and useful) models, feature selection should be performed in gene expression datasets due to the redundancy and irrelevance of many of the features in these high dimensional datasets. It is highly important that the testing dataset be independent and not involved in the feature selection or model training to get accurate estimates of model performance. Following these guidelines will enable researchers to build on the progress made applying state-of-the-art ML methods to precision medicine in AUD.

## Summary

This Review tabulated and summarized the studies on peripheral RNA biomarkers for AUD. Signatures of chronic and acute alcohol use could be detected in the peripheral transcriptome for both coding and non-coding RNAs, and some of these gene expression responses were detected within relatively short time periods after alcohol or stress cue exposures (60–90 min). Most of the studies did not include a measure of biomarker performance which limits the ability to determine the usefulness of RNAs to serve as biomarkers for AUD. Based on the studies that did include a measure of biomarker performance, the ability of RNA levels to serve as diagnostic biomarkers and predict AUD or other alcohol-related phenotypes was generally good (AUC > 0.70). To facilitate AUD biomarker research, future studies should include a measure of biomarker performance. There is a scarcity of studies investigating the ability of RNAs to serve as other types of biomarkers. While there are existing means for diagnosing AUD, there are no clinical tools for predicting at-risk individuals, stratifying patient groups, prioritizing optimal treatments, or monitoring treatment response. Therefore, this is an unmet healthcare need and future work should include longitudinal studies with repeated sampling to evaluate this potential. It will also be important to see if integrating data across -omics platforms results in an improved biomarker performance using ML approaches. There were no studies assessing alcohol’s effects on some categories of regulatory non-coding RNAs (lncRNAs and piRNAs), so these represent additional unexplored avenues of research for future studies.

## Conclusion

These studies suggest that there is an AUD-related signature in peripheral tissue that holds diagnostic utility. Based on these findings, we are confident that -omics technologies and systems biology approaches will identify clinically useful biomarkers for AUD, however, establishing models that link mechanisms and biomarkers is likely to be a challenge for the coming decades of basic and clinical research. The identified RNAs in these studies will require further evaluation and validation to find markers with high specificity, low cost, and ease-of-use in routine diagnostic laboratories. Laboratory analysis of RNA has been facilitated by PCR methodologies now available in many diagnostic centers because of the COVID-19 pandemic.

## Author contributions

LF: conceptualization, data curation, investigation, methodology, project administration, visualization, writing – original draft, review, and editing. RDM: supervision and writing – review and editing. ROM: funding acquisition, resources, supervision, and writing – review and editing. All authors contributed to the article and approved the submitted version.

## Footnotes

1
https://medicalfuturist.com/fda-approved-ai-based-algorithms/
