# Supplementary material for: RNA biomarkers for alcohol use disorder
Source: Front Mol Neurosci. 2022 Nov 4;15:1032362. doi: 10.3389/fnmol.2022.1032362 (PMC9673015; doi:10.3389/fnmol.2022.1032362)
Supplement: Supplementary file 1 [file Table_1.docx]

Supplementary Material

**Supplementary Table 1.** **Gene Expression Profiles Sourced from Peripheral Tissues Inform Brain Diseases: Examples.**

| **Behavioral Disorder or Psychiatric Illness** | **References** |
| --- | --- |
| Substance Use Disorders | nicotine (Philibert et al., 2007); opioid (Xu et al., 2020); cocaine (Fries et al., 2018) |
| Autism | (Hicks et al., 2018, Lit et al., 2012, Lombardo et al., 2018, Nishimura et al., 2007, Pramparo et al., 2015, Tylee et al., 2017, Yao et al., 2021) |
| Depression | (Cattaneo et al., 2020, Cho et al., 2019, Clark et al., 2020, Frye et al., 2015, Grant et al., 2022, Hepgul et al., 2016, Hori et al., 2018, Jani et al., 2015, Keri et al., 2014, Le-Niculescu et al., 2021, Maffioletti et al., 2016, Mostafavi et al., 2014, Nohr et al., 2021, Redei et al., 2014, van der Zee et al., 2022, Wittenberg et al., 2020, Cook et al., 2019, Gomez Rueda and Bustillo, 2022, Mamdani et al., 2022) |
| ADHD And Emotional/Behavioral Problems | (Hess et al., 2020, Lorenzo et al., 2018, Sanchez-Mora et al., 2019, Tian et al., 2012, Ota et al., 2020) |
| Bipolar Disorder | (Beech et al., 2014, Eugene et al., 2018, Hashimoto, 2018, Krebs et al., 2020, Le-Niculescu et al., 2009, Le-Niculescu et al., 2021, Lowthert et al., 2012, Maffioletti et al., 2016, Munkholm et al., 2015, Salvetat et al., 2022, Tsuang et al., 2005, Song et al., 2021) |
| Schizophrenia | (Breen et al., 2016, Chen et al., 2020, de Jong et al., 2012, Gasso et al., 2017, Gilabert-Juan et al., 2019, Glatt et al., 2005, He et al., 2012, Sershen et al., 2021, Song et al., 2021, Takahashi et al., 2010, Tsuang et al., 2005, Wagh et al., 2021, Walton et al., 2016, Zhu et al., 2021) |
| Obsessive Compulsive Disorder | (Wang et al., 2018, Song et al., 2021) |
| PTSD / Other Stress States | (Balakathiresan et al., 2014, Daskalakis et al., 2014, Dean et al., 2020, Garrett et al., 2021, Le-Niculescu et al., 2019, Maguire et al., 2021, Papassotiropoulos et al., 2013, Snijders et al., 2020, van Heerden et al., 2009, Yang et al., 2013) |
| Neurodegenerative Diseases | (Chiricosta et al., 2022, Dobromyslin et al., 2022, Hensman Moss et al., 2017, Iturria-Medina et al., 2020, Lee and Lee, 2020, Milanesi et al., 2021, Niculescu et al., 2020, Ochi et al., 2020, Sh et al., 2021, Song et al., 2021, Borovecki et al., 2005, Falchetti et al., 2020, Mina et al., 2016, Valentine et al., 2019) |

**
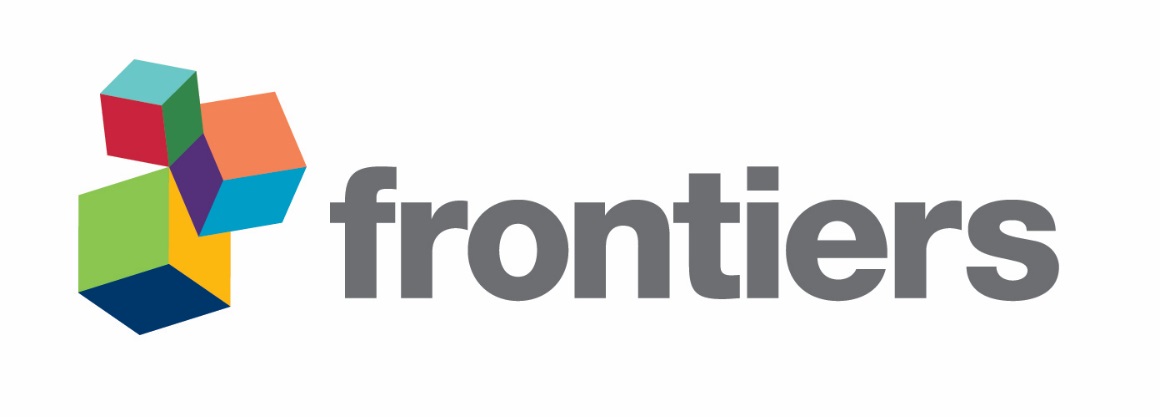
**

BALAKATHIRESAN, N. S., CHANDRAN, R., BHOMIA, M., JIA, M., LI, H. & MAHESHWARI, R. K. 2014. Serum and amygdala microRNA signatures of posttraumatic stress: fear correlation and biomarker potential. *J Psychiatr Res,* 57**,** 65-73.

BEECH, R. D., LEFFERT, J. J., LIN, A., SYLVIA, L. G., UMLAUF, S., MANE, S., ZHAO, H., BOWDEN, C., CALABRESE, J. R., FRIEDMAN, E. S., KETTER, T. A., IOSIFESCU, D. V., REILLY-HARRINGTON, N. A., OSTACHER, M., THASE, M. E. & NIERENBERG, A. 2014. Gene-expression differences in peripheral blood between lithium responders and non-responders in the Lithium Treatment-Moderate dose Use Study (LiTMUS). *Pharmacogenomics J,* 14**,** 182-91.

BOROVECKI, F., LOVRECIC, L., ZHOU, J., JEONG, H., THEN, F., ROSAS, H. D., HERSCH, S. M., HOGARTH, P., BOUZOU, B., JENSEN, R. V. & KRAINC, D. 2005. Genome-wide expression profiling of human blood reveals biomarkers for Huntington's disease. *Proc Natl Acad Sci U S A,* 102**,** 11023-8.

BREEN, M. S., UHLMANN, A., NDAY, C. M., GLATT, S. J., MITT, M., METSALPU, A., STEIN, D. J. & ILLING, N. 2016. Candidate gene networks and blood biomarkers of methamphetamine-associated psychosis: an integrative RNA-sequencing report. *Transl Psychiatry,* 6**,** e802.

CATTANEO, A., FERRARI, C., TURNER, L., MARIANI, N., ENACHE, D., HASTINGS, C., KOSE, M., LOMBARDO, G., MCLAUGHLIN, A. P., NETTIS, M. A., NIKKHESLAT, N., SFORZINI, L., WORRELL, C., ZAJKOWSKA, Z., CATTANE, N., LOPIZZO, N., MAZZELLI, M., POINTON, L., COWEN, P. J., CAVANAGH, J., HARRISON, N. A., DE BOER, P., JONES, D., DREVETS, W. C., MONDELLI, V., BULLMORE, E. T., NEUROIMMUNOLOGY OF MOOD, D., ALZHEIMER'S DISEASE, C. & PARIANTE, C. M. 2020. Whole-blood expression of inflammasome- and glucocorticoid-related mRNAs correctly separates treatment-resistant depressed patients from drug-free and responsive patients in the BIODEP study. *Transl Psychiatry,* 10**,** 232.

CHEN, J., ZANG, Z., BRAUN, U., SCHWARZ, K., HARNEIT, A., KREMER, T., MA, R., SCHWEIGER, J., MOESSNANG, C., GEIGER, L., CAO, H., DEGENHARDT, F., NOTHEN, M. M., TOST, H., MEYER-LINDENBERG, A. & SCHWARZ, E. 2020. Association of a Reproducible Epigenetic Risk Profile for Schizophrenia With Brain Methylation and Function. *JAMA Psychiatry,* 77**,** 628-636.

CHIRICOSTA, L., D'ANGIOLINI, S., GUGLIANDOLO, A. & MAZZON, E. 2022. Artificial Intelligence Predictor for Alzheimer's Disease Trained on Blood Transcriptome: The Role of Oxidative Stress. *Int J Mol Sci,* 23.

CHO, J. H., IRWIN, M. R., EISENBERGER, N. I., LAMKIN, D. M. & COLE, S. W. 2019. Transcriptomic predictors of inflammation-induced depressed mood. *Neuropsychopharmacology,* 44**,** 923-929.

CLARK, S. L., HATTAB, M. W., CHAN, R. F., SHABALIN, A. A., HAN, L. K. M., ZHAO, M., SMIT, J. H., JANSEN, R., MILANESCHI, Y., XIE, L. Y., VAN GROOTHEEST, G., PENNINX, B., ABERG, K. A. & VAN DEN OORD, E. 2020. A methylation study of long-term depression risk. *Mol Psychiatry,* 25**,** 1334-1343.

COOK, I. A., CONGDON, E., KRANTZ, D. E., HUNTER, A. M., COPPOLA, G., HAMILTON, S. P. & LEUCHTER, A. F. 2019. Time Course of Changes in Peripheral Blood Gene Expression During Medication Treatment for Major Depressive Disorder. *Front Genet,* 10**,** 870.

DASKALAKIS, N. P., COHEN, H., CAI, G., BUXBAUM, J. D. & YEHUDA, R. 2014. Expression profiling associates blood and brain glucocorticoid receptor signaling with trauma-related individual differences in both sexes. *Proc Natl Acad Sci U S A,* 111**,** 13529-34.

DE JONG, S., BOKS, M. P., FULLER, T. F., STRENGMAN, E., JANSON, E., DE KOVEL, C. G., ORI, A. P., VI, N., MULDER, F., BLOM, J. D., GLENTHØJ, B., SCHUBART, C. D., CAHN, W., KAHN, R. S., HORVATH, S. & OPHOFF, R. A. 2012. A gene co-expression network in whole blood of schizophrenia patients is independent of antipsychotic-use and enriched for brain-expressed genes. *PLoS One,* 7**,** e39498.

DEAN, K. R., HAMMAMIEH, R., MELLON, S. H., ABU-AMARA, D., FLORY, J. D., GUFFANTI, G., WANG, K., DAIGLE, B. J., JR., GAUTAM, A., LEE, I., YANG, R., ALMLI, L. M., BERSANI, F. S., CHAKRABORTY, N., DONOHUE, D., KERLEY, K., KIM, T. K., LASKA, E., YOUNG LEE, M., LINDQVIST, D., LORI, A., LU, L., MISGANAW, B., MUHIE, S., NEWMAN, J., PRICE, N. D., QIN, S., REUS, V. I., SIEGEL, C., SOMVANSHI, P. R., THAKUR, G. S., ZHOU, Y., CONSORTIUM, P. S. B., HOOD, L., RESSLER, K. J., WOLKOWITZ, O. M., YEHUDA, R., JETT, M., DOYLE, F. J., 3RD & MARMAR, C. 2020. Multi-omic biomarker identification and validation for diagnosing warzone-related post-traumatic stress disorder. *Mol Psychiatry,* 25**,** 3337-3349.

DOBROMYSLIN, V. I., MEGHERBI, D. B. & ALZHEIMER'S DISEASE NEUROIMAGING, I. 2022. Augmenting Imaging Biomarker Performance with Blood-Based Gene Expression Levels for Predicting Alzheimer's Disease Progression. *J Alzheimers Dis,* 87**,** 583-594.

EUGENE, A. R., MASIAK, J. & EUGENE, B. 2018. Predicting lithium treatment response in bipolar patients using gender-specific gene expression biomarkers and machine learning. *F1000Res,* 7**,** 474.

FALCHETTI, M., PREDIGER, R. D. & ZANOTTO-FILHO, A. 2020. Classification algorithms applied to blood-based transcriptome meta-analysis to predict idiopathic Parkinson's disease. *Comput Biol Med,* 124**,** 103925.

FRIES, G. R., KHAN, S., STAMATOVICH, S., DYUKOVA, E., WALSS-BASS, C., LANE, S. D., SCHMITZ, J. M. & WARDLE, M. C. 2018. Anhedonia in cocaine use disorder is associated with inflammatory gene expression. *PLoS One,* 13**,** e0207231.

FRYE, M. A., NASSAN, M., JENKINS, G. D., KUNG, S., VELDIC, M., PALMER, B. A., FEEDER, S. E., TYE, S. J., CHOI, D. S. & BIERNACKA, J. M. 2015. Feasibility of investigating differential proteomic expression in depression: implications for biomarker development in mood disorders. *Transl Psychiatry,* 5**,** e689.

GARRETT, M. E., QIN, X. J., MEHTA, D., DENNIS, M. F., MARX, C. E., GRANT, G. A., WORKGROUP, V. A. M.-A. M., INITIATIVE, P., INJURY, TRAUMATIC STRESS CLINICAL, C., PSYCHIATRIC GENOMICS CONSORTIUM, P. G., STEIN, M. B., KIMBREL, N. A., BECKHAM, J. C., HAUSER, M. A. & ASHLEY-KOCH, A. E. 2021. Gene Expression Analysis in Three Posttraumatic Stress Disorder Cohorts Implicates Inflammation and Innate Immunity Pathways and Uncovers Shared Genetic Risk With Major Depressive Disorder. *Front Neurosci,* 15**,** 678548.

GASSO, P., MAS, S., RODRIGUEZ, N., BOLOC, D., GARCIA-CERRO, S., BERNARDO, M., LAFUENTE, A. & PARELLADA, E. 2017. Microarray gene-expression study in fibroblast and lymphoblastoid cell lines from antipsychotic-naive first-episode schizophrenia patients. *J Psychiatr Res,* 95**,** 91-101.

GILABERT-JUAN, J., LOPEZ-CAMPOS, G., SEBASTIA-ORTEGA, N., GUARA-CIURANA, S., RUSO-JULVE, F., PRIETO, C., CRESPO-FACORRO, B., SANJUAN, J. & MOLTO, M. D. 2019. Time dependent expression of the blood biomarkers EIF2D and TOX in patients with schizophrenia. *Brain Behav Immun,* 80**,** 909-915.

GLATT, S. J., EVERALL, I. P., KREMEN, W. S., CORBEIL, J., SASIK, R., KHANLOU, N., HAN, M., LIEW, C. C. & TSUANG, M. T. 2005. Comparative gene expression analysis of blood and brain provides concurrent validation of SELENBP1 up-regulation in schizophrenia. *Proc Natl Acad Sci U S A,* 102**,** 15533-8.

GOMEZ RUEDA, H. & BUSTILLO, J. 2022. Brain differential gene expression and blood cross-validation of a molecular signature of patients with major depressive disorder. *Psychiatr Genet,* 32**,** 105-115.

GRANT, C. W., BARRETO, E. F., KUMAR, R., KADDURAH-DAOUK, R., SKIME, M., MAYES, T., CARMODY, T., BIERNACKA, J., WANG, L., WEINSHILBOUM, R., TRIVEDI, M. H., BOBO, W. V., CROARKIN, P. E. & ATHREYA, A. P. 2022. Multi-Omics Characterization of Early- and Adult-Onset Major Depressive Disorder. *J Pers Med,* 12.

HASHIMOTO, K. 2018. Metabolomics of Major Depressive Disorder and Bipolar Disorder: Overview and Future Perspective. *Adv Clin Chem,* 84**,** 81-99.

HE, Y., YU, Z., GIEGLING, I., XIE, L., HARTMANN, A. M., PREHN, C., ADAMSKI, J., KAHN, R., LI, Y., ILLIG, T., WANG-SATTLER, R. & RUJESCU, D. 2012. Schizophrenia shows a unique metabolomics signature in plasma. *Transl Psychiatry,* 2**,** e149.

HENSMAN MOSS, D. J., FLOWER, M. D., LO, K. K., MILLER, J. R., VAN OMMEN, G. B., T HOEN, P. A., STONE, T. C., GUINEE, A., LANGBEHN, D. R., JONES, L., PLAGNOL, V., VAN ROON-MOM, W. M., HOLMANS, P. & TABRIZI, S. J. 2017. Huntington's disease blood and brain show a common gene expression pattern and share an immune signature with Alzheimer's disease. *Sci Rep,* 7**,** 44849.

HEPGUL, N., CATTANEO, A., AGARWAL, K., BARALDI, S., BORSINI, A., BUFALINO, C., FORTON, D. M., MONDELLI, V., NIKKHESLAT, N., LOPIZZO, N., RIVA, M. A., RUSSELL, A., HOTOPF, M. & PARIANTE, C. M. 2016. Transcriptomics in Interferon-alpha-Treated Patients Identifies Inflammation-, Neuroplasticity- and Oxidative Stress-Related Signatures as Predictors and Correlates of Depression. *Neuropsychopharmacology,* 41**,** 2502-11.

HESS, J. L., NGUYEN, N. H., SUBEN, J., MEATH, R. M., ALBERT, A. B., VAN ORMAN, S., ANDERS, K. M., FORKEN, P. J., ROE, C. A., SCHULZE, T. G., FARAONE, S. V. & GLATT, S. J. 2020. Gene co-expression networks in peripheral blood capture dimensional measures of emotional and behavioral problems from the Child Behavior Checklist (CBCL). *Transl Psychiatry,* 10**,** 328.

HICKS, S. D., RAJAN, A. T., WAGNER, K. E., BARNS, S., CARPENTER, R. L. & MIDDLETON, F. A. 2018. Validation of a Salivary RNA Test for Childhood Autism Spectrum Disorder. *Front Genet,* 9**,** 534.

HORI, H., NAKAMURA, S., YOSHIDA, F., TERAISHI, T., SASAYAMA, D., OTA, M., HATTORI, K., KIM, Y., HIGUCHI, T. & KUNUGI, H. 2018. Integrated profiling of phenotype and blood transcriptome for stress vulnerability and depression. *J Psychiatr Res,* 104**,** 202-210.

ITURRIA-MEDINA, Y., KHAN, A. F., ADEWALE, Q., SHIRAZI, A. H. & ALZHEIMER'S DISEASE NEUROIMAGING, I. 2020. Blood and brain gene expression trajectories mirror neuropathology and clinical deterioration in neurodegeneration. *Brain,* 143**,** 661-673.

JANI, B. D., MCLEAN, G., NICHOLL, B. I., BARRY, S. J., SATTAR, N., MAIR, F. S. & CAVANAGH, J. 2015. Risk assessment and predicting outcomes in patients with depressive symptoms: a review of potential role of peripheral blood based biomarkers. *Front Hum Neurosci,* 9**,** 18.

KERI, S., SZABO, C. & KELEMEN, O. 2014. Blood biomarkers of depression track clinical changes during cognitive-behavioral therapy. *J Affect Disord,* 164**,** 118-22.

KREBS, C. E., ORI, A. P. S., VREEKER, A., WU, T., CANTOR, R. M., BOKS, M. P. M., KAHN, R. S., OLDE LOOHUIS, L. M. & OPHOFF, R. A. 2020. Whole blood transcriptome analysis in bipolar disorder reveals strong lithium effect. *Psychol Med,* 50**,** 2575-2586.

LE-NICULESCU, H., KURIAN, S. M., YEHYAWI, N., DIKE, C., PATEL, S. D., EDENBERG, H. J., TSUANG, M. T., SALOMON, D. R., NURNBERGER, J. I., JR. & NICULESCU, A. B. 2009. Identifying blood biomarkers for mood disorders using convergent functional genomics. *Mol Psychiatry,* 14**,** 156-74.

LE-NICULESCU, H., ROSEBERRY, K., GILL, S. S., LEVEY, D. F., PHALEN, P. L., MULLEN, J., WILLIAMS, A., BHAIRO, S., VOEGTLINE, T., DAVIS, H., SHEKHAR, A., KURIAN, S. M. & NICULESCU, A. B. 2021. Precision medicine for mood disorders: objective assessment, risk prediction, pharmacogenomics, and repurposed drugs. *Mol Psychiatry,* 26**,** 2776-2804.

LE-NICULESCU, H., ROSEBERRY, K., LEVEY, D. F., ROGERS, J., KOSARY, K., PRABHA, S., JONES, T., JUDD, S., MCCORMICK, M. A., WESSEL, A. R., WILLIAMS, A., PHALEN, P. L., MAMDANI, F., SEQUEIRA, A., KURIAN, S. M. & NICULESCU, A. B. 2019. Towards precision medicine for stress disorders: diagnostic biomarkers and targeted drugs. *Mol Psychiatry*.

LEE, T. & LEE, H. 2020. Prediction of Alzheimer's disease using blood gene expression data. *Sci Rep,* 10**,** 3485.

LIT, L., SHARP, F. R., BERTOGLIO, K., STAMOVA, B., ANDER, B. P., SOSSONG, A. D. & HENDREN, R. L. 2012. Gene expression in blood is associated with risperidone response in children with autism spectrum disorders. *Pharmacogenomics J,* 12**,** 368-71.

LOMBARDO, M. V., PRAMPARO, T., GAZESTANI, V., WARRIER, V., BETHLEHEM, R. A. I., CARTER BARNES, C., LOPEZ, L., LEWIS, N. E., EYLER, L., PIERCE, K. & COURCHESNE, E. 2018. Large-scale associations between the leukocyte transcriptome and BOLD responses to speech differ in autism early language outcome subtypes. *Nat Neurosci,* 21**,** 1680-1688.

LORENZO, G., BRAUN, J., MUNOZ, G., CASAREJOS, M. J., BAZAN, E. & JIMENEZ-ESCRIG, A. 2018. RNA-Seq blood transcriptome profiling in familial attention deficit and hyperactivity disorder (ADHD). *Psychiatry Res,* 270**,** 544-546.

LOWTHERT, L., LEFFERT, J., LIN, A., UMLAUF, S., MALONEY, K., MURALIDHARAN, A., LORBERG, B., MANE, S., ZHAO, H., SINHA, R., BHAGWAGAR, Z. & BEECH, R. 2012. Increased ratio of anti-apoptotic to pro-apoptotic Bcl2 gene-family members in lithium-responders one month after treatment initiation. *Biol Mood Anxiety Disord,* 2**,** 15.

MAFFIOLETTI, E., CATTANEO, A., ROSSO, G., MAINA, G., MAJ, C., GENNARELLI, M., TARDITO, D. & BOCCHIO-CHIAVETTO, L. 2016. Peripheral whole blood microRNA alterations in major depression and bipolar disorder. *J Affect Disord,* 200**,** 250-8.

MAGUIRE, D., WATT, J., ARMOUR, C., MILANAK, M., LAGDON, S., LAMONT, J. V., KURTH, M. J., FITZGERALD, P., MOORE, T. & RUDDOCK, M. W. 2021. Post-traumatic stress disorder: A biopsychosocial case-control study investigating peripheral blood protein biomarkers. *Biomarkers in Neuropsychiatry,* 5**,** 100042.

MAMDANI, F., WEBER, M. D., BUNNEY, B., BURKE, K., CARTAGENA, P., WALSH, D., LEE, F. S., BARCHAS, J., SCHATZBERG, A. F., MYERS, R. M., WATSON, S. J., AKIL, H., VAWTER, M. P., BUNNEY, W. E. & SEQUEIRA, A. 2022. Identification of potential blood biomarkers associated with suicide in major depressive disorder. *Transl Psychiatry,* 12**,** 159.

MILANESI, E., DOBRE, M., CUCOS, C. A., ROJO, A. I., JIMENEZ-VILLEGAS, J., CAPETILLO-ZARATE, E., MATUTE, C., PINOL-RIPOLL, G., MANDA, G. & CUADRADO, A. 2021. Whole Blood Expression Pattern of Inflammation and Redox Genes in Mild Alzheimer's Disease. *J Inflamm Res,* 14**,** 6085-6102.

MINA, E., VAN ROON-MOM, W., HETTNE, K., VAN ZWET, E., GOEMAN, J., NERI, C., P, A. C. T. H., MONS, B. & ROOS, M. 2016. Common disease signatures from gene expression analysis in Huntington's disease human blood and brain. *Orphanet J Rare Dis,* 11**,** 97.

MOSTAFAVI, S., BATTLE, A., ZHU, X., POTASH, J. B., WEISSMAN, M. M., SHI, J., BECKMAN, K., HAUDENSCHILD, C., MCCORMICK, C., MEI, R., GAMEROFF, M. J., GINDES, H., ADAMS, P., GOES, F. S., MONDIMORE, F. M., MACKINNON, D. F., NOTES, L., SCHWEIZER, B., FURMAN, D., MONTGOMERY, S. B., URBAN, A. E., KOLLER, D. & LEVINSON, D. F. 2014. Type I interferon signaling genes in recurrent major depression: increased expression detected by whole-blood RNA sequencing. *Mol Psychiatry,* 19**,** 1267-74.

MUNKHOLM, K., PEIJS, L., VINBERG, M. & KESSING, L. V. 2015. A composite peripheral blood gene expression measure as a potential diagnostic biomarker in bipolar disorder. *Transl Psychiatry,* 5**,** e614.

NICULESCU, A. B., LE-NICULESCU, H., ROSEBERRY, K., WANG, S., HART, J., KAUR, A., ROBERTSON, H., JONES, T., STRASBURGER, A., WILLIAMS, A., KURIAN, S. M., LAMB, B., SHEKHAR, A., LAHIRI, D. K. & SAYKIN, A. J. 2020. Blood biomarkers for memory: toward early detection of risk for Alzheimer disease, pharmacogenomics, and repurposed drugs. *Mol Psychiatry,* 25**,** 1651-1672.

NISHIMURA, Y., MARTIN, C. L., VAZQUEZ-LOPEZ, A., SPENCE, S. J., ALVAREZ-RETUERTO, A. I., SIGMAN, M., STEINDLER, C., PELLEGRINI, S., SCHANEN, N. C., WARREN, S. T. & GESCHWIND, D. H. 2007. Genome-wide expression profiling of lymphoblastoid cell lines distinguishes different forms of autism and reveals shared pathways. *Hum Mol Genet,* 16**,** 1682-98.

NOHR, A. K., LINDOW, M., FORSINGDAL, A., DEMHARTER, S., NIELSEN, T., BULLER, R., MOLTKE, I., VITEZIC, M. & ALBRECHTSEN, A. 2021. A large-scale genome-wide gene expression analysis in peripheral blood identifies very few differentially expressed genes related to antidepressant treatment and response in patients with major depressive disorder. *Neuropsychopharmacology,* 46**,** 1324-1332.

OCHI, S., IGA, J. I., FUNAHASHI, Y., YOSHINO, Y., YAMAZAKI, K., KUMON, H., MORI, H., OZAKI, Y., MORI, T. & UENO, S. I. 2020. Identifying Blood Transcriptome Biomarkers of Alzheimer's Disease Using Transgenic Mice. *Mol Neurobiol,* 57**,** 4941-4951.

OTA, V. K., SANTORO, M. L., SPINDOLA, L. M., PAN, P. M., SIMABUCURO, A., XAVIER, G., VIEIRA-FONSECA, T., ZANARDO, E. A., DOS SANTOS, F. R. C., SCHAFER, J. L., KULIKOWSKI, L. D., GALANTE, P. A. F., ASPRINO, P. F., BRIETZKE, E., GRASSI-OLIVEIRA, R., ROHDE, L. A., MIGUEL, E. C., GADELHA, A., MARI, J. J., BRESSAN, R. A., SALUM, G. A. & BELANGERO, S. I. 2020. Gene expression changes associated with trajectories of psychopathology in a longitudinal cohort of children and adolescents. *Transl Psychiatry,* 10**,** 99.

PAPASSOTIROPOULOS, A., GERHARDS, C., HECK, A., ACKERMANN, S., AERNI, A., SCHICKTANZ, N., AUSCHRA, B., DEMOUGIN, P., MUMME, E., ELBERT, T., ERTL, V., GSCHWIND, L., HANSER, E., HUYNH, K. D., JESSEN, F., KOLASSA, I. T., MILNIK, A., PAGANETTI, P., SPALEK, K., VOGLER, C., MUHS, A., PFEIFER, A. & DE QUERVAIN, D. J. 2013. Human genome-guided identification of memory-modulating drugs. *Proc Natl Acad Sci U S A,* 110**,** E4369-74.

PHILIBERT, R. A., RYU, G. Y., YOON, J. G., SANDHU, H., HOLLENBECK, N., GUNTER, T., BARKHURST, A., ADAMS, W. & MADAN, A. 2007. Transcriptional profiling of subjects from the Iowa adoption studies. *Am J Med Genet B Neuropsychiatr Genet,* 144B**,** 683-90.

PRAMPARO, T., PIERCE, K., LOMBARDO, M. V., CARTER BARNES, C., MARINERO, S., AHRENS-BARBEAU, C., MURRAY, S. S., LOPEZ, L., XU, R. & COURCHESNE, E. 2015. Prediction of autism by translation and immune/inflammation coexpressed genes in toddlers from pediatric community practices. *JAMA Psychiatry,* 72**,** 386-94.

REDEI, E. E., ANDRUS, B. M., KWASNY, M. J., SEOK, J., CAI, X., HO, J. & MOHR, D. C. 2014. Blood transcriptomic biomarkers in adult primary care patients with major depressive disorder undergoing cognitive behavioral therapy. *Transl Psychiatry,* 4**,** e442.

SALVETAT, N., CHECA-ROBLES, F. J., PATEL, V., CAYZAC, C., DUBUC, B., CHIMIENTI, F., ABRAHAM, J. D., DUPRE, P., VETTER, D., MEREUZE, S., LANG, J. P., KUPFER, D. J., COURTET, P. & WEISSMANN, D. 2022. A game changer for bipolar disorder diagnosis using RNA editing-based biomarkers. *Transl Psychiatry,* 12**,** 182.

SANCHEZ-MORA, C., SOLER ARTIGAS, M., GARCIA-MARTINEZ, I., PAGEROLS, M., ROVIRA, P., RICHARTE, V., CORRALES, M., FADEUILHE, C., PADILLA, N., DE LA CRUZ, X., FRANKE, B., ARIAS-VASQUEZ, A., CASAS, M., RAMOS-QUIROGA, J. A. & RIBASES, M. 2019. Epigenetic signature for attention-deficit/hyperactivity disorder: identification of miR-26b-5p, miR-185-5p, and miR-191-5p as potential biomarkers in peripheral blood mononuclear cells. *Neuropsychopharmacology,* 44**,** 890-897.

SERSHEN, H., GUIDOTTI, A., AUTA, J., DRNEVICH, J., GRAYSON, D. R., VELDIC, M., MEYERS, J., YOUSEFF, M., ZHUBI, A., FAUROT, K., WU, R., ZHAO, J., JIN, H., LAJTHA, A., DAVIS, J. M. & SMITH, R. C. 2021. Gene expression of methylation cycle and related genes in lymphocytes and brain of patients with schizophrenia and non-psychotic controls. *Biomarkers in Neuropsychiatry,* 5**,** 100038.

SH, Y., LIU, B., ZHANG, J., ZHOU, Y., HU, Z. & ZHANG, X. 2021. Application of Artificial Intelligence Modeling Technology Based on Fluid Biopsy to Diagnose Alzheimer's Disease. *Front Aging Neurosci,* 13**,** 768229.

SNIJDERS, C., MAIHOFER, A. X., RATANATHARATHORN, A., BAKER, D. G., BOKS, M. P., GEUZE, E., JAIN, S., KESSLER, R. C., PISHVA, E., RISBROUGH, V. B., STEIN, M. B., URSANO, R. J., VERMETTEN, E., VINKERS, C. H., CONSORTIUM, P. P. E., SMITH, A. K., UDDIN, M., RUTTEN, B. P. F. & NIEVERGELT, C. M. 2020. Longitudinal epigenome-wide association studies of three male military cohorts reveal multiple CpG sites associated with post-traumatic stress disorder. *Clin Epigenetics,* 12**,** 11.

SONG, W., WANG, W., LIU, Z., CAI, W., YU, S., ZHAO, M. & LIN, G. N. 2021. A Comprehensive Evaluation of Cross-Omics Blood-Based Biomarkers for Neuropsychiatric Disorders. *J Pers Med,* 11.

TAKAHASHI, M., HAYASHI, H., WATANABE, Y., SAWAMURA, K., FUKUI, N., WATANABE, J., KITAJIMA, T., YAMANOUCHI, Y., IWATA, N., MIZUKAMI, K., HORI, T., SHIMODA, K., UJIKE, H., OZAKI, N., IIJIMA, K., TAKEMURA, K., AOSHIMA, H. & SOMEYA, T. 2010. Diagnostic classification of schizophrenia by neural network analysis of blood-based gene expression signatures. *Schizophr Res,* 119**,** 210-8.

TIAN, Y., STAMOVA, B., ANDER, B. P., JICKLING, G. C., GUNTHER, J. R., CORBETT, B. A., BOS-VENEMAN, N. G., HOEKSTRA, P. J., SCHWEITZER, J. B. & SHARP, F. R. 2012. Correlations of gene expression with ratings of inattention and hyperactivity/impulsivity in Tourette syndrome: a pilot study. *BMC Med Genomics,* 5**,** 49.

TSUANG, M. T., NOSSOVA, N., YAGER, T., TSUANG, M. M., GUO, S. C., SHYU, K. G., GLATT, S. J. & LIEW, C. C. 2005. Assessing the validity of blood-based gene expression profiles for the classification of schizophrenia and bipolar disorder: a preliminary report. *Am J Med Genet B Neuropsychiatr Genet,* 133B**,** 1-5.

TYLEE, D. S., HESS, J. L., QUINN, T. P., BARVE, R., HUANG, H., ZHANG-JAMES, Y., CHANG, J., STAMOVA, B. S., SHARP, F. R., HERTZ-PICCIOTTO, I., FARAONE, S. V., KONG, S. W. & GLATT, S. J. 2017. Blood transcriptomic comparison of individuals with and without autism spectrum disorder: A combined-samples mega-analysis. *Am J Med Genet B Neuropsychiatr Genet,* 174**,** 181-201.

VALENTINE, M. N. Z., HASHIMOTO, K., FUKUHARA, T., SAIKI, S., ISHIKAWA, K. I., HATTORI, N. & CARNINCI, P. 2019. Multi-year whole-blood transcriptome data for the study of onset and progression of Parkinson's Disease. *Sci Data,* 6**,** 20.

VAN DER ZEE, Y. Y., EIJSSEN, L. M. T., MEWS, P., RAMAKRISHNAN, A., ALVAREZ, K., LARDNER, C. K., CATES, H. M., WALKER, D. M., TORRES-BERRIO, A., BROWNE, C. J., CUNNINGHAM, A., CATHOMAS, F., KRONMAN, H., PARISE, E. M., DE NIJS, L., SHEN, L., MURROUGH, J. W., RUTTEN, B. P. F., NESTLER, E. J. & ISSLER, O. 2022. Blood miR-144-3p: a novel diagnostic and therapeutic tool for depression. *Mol Psychiatry*.

VAN HEERDEN, J. H., CONESA, A., STEIN, D. J., MONTANER, D., RUSSELL, V. & ILLING, N. 2009. Parallel changes in gene expression in peripheral blood mononuclear cells and the brain after maternal separation in the mouse. *BMC Res Notes,* 2**,** 195.

WAGH, V. V., VYAS, P., AGRAWAL, S., PACHPOR, T. A., PARALIKAR, V. & KHARE, S. P. 2021. Peripheral Blood-Based Gene Expression Studies in Schizophrenia: A Systematic Review. *Front Genet,* 12**,** 736483.

WALTON, E., HASS, J., LIU, J., ROFFMAN, J. L., BERNARDONI, F., ROESSNER, V., KIRSCH, M., SCHACKERT, G., CALHOUN, V. & EHRLICH, S. 2016. Correspondence of DNA Methylation Between Blood and Brain Tissue and Its Application to Schizophrenia Research. *Schizophr Bull,* 42**,** 406-14.

WANG, Y., CHENG, C., ZHANG, Z., WANG, J., WANG, Y., LI, X., GAO, R., WANG, Z., FANG, Y., WANG, J., WANG, M., FAN, Q., PERIYA, S., ZHANG, H., TSUANG, M. T. & LIEW, C. C. 2018. Blood-based dynamic genomic signature for obsessive-compulsive disorder. *Am J Med Genet B Neuropsychiatr Genet,* 177**,** 709-716.

WITTENBERG, G. M., GREENE, J., VERTES, P. E., DREVETS, W. C. & BULLMORE, E. T. 2020. Major Depressive Disorder Is Associated With Differential Expression of Innate Immune and Neutrophil-Related Gene Networks in Peripheral Blood: A Quantitative Review of Whole-Genome Transcriptional Data From Case-Control Studies. *Biol Psychiatry,* 88**,** 625-637.

XU, W., ZHAO, M., LIN, Z., LIU, H., MA, H., HONG, Q., GUI, D., FENG, J., LIU, Y., ZHOU, W. & LIU, H. 2020. Increased expression of plasma hsa-miR-181a in male patients with heroin addiction use disorder. *J Clin Lab Anal,* 34**,** e23486.

YANG, R., DAIGLE, B. J., JR., MUHIE, S. Y., HAMMAMIEH, R., JETT, M., PETZOLD, L. & DOYLE, F. J., 3RD 2013. Core modular blood and brain biomarkers in social defeat mouse model for post traumatic stress disorder. *BMC Syst Biol,* 7**,** 80.

YAO, F., ZHANG, K., FENG, C., GAO, Y., SHEN, L., LIU, X. & NI, J. 2021. Protein Biomarkers of Autism Spectrum Disorder Identified by Computational and Experimental Methods. *Front Psychiatry,* 12**,** 554621.

ZHU, L., WU, X., XU, B., ZHAO, Z., YANG, J., LONG, J. & SU, L. 2021. The machine learning algorithm for the diagnosis of schizophrenia on the basis of gene expression in peripheral blood. *Neurosci Lett,* 745**,** 135596.
